# Ultrahigh-speed laser drilling of transparent materials via transient electronic excitation

**DOI:** 10.1126/sciadv.adv4436

**Published:** 2025-06-11

**Authors:** Yanming Zhang, Takumi Koike, Reina Yoshizaki, Guoqi Ren, Akihiro Shibata, Sota Kiriake, Ryota Hasegawa, Ikuo Nagasawa, Keisuke Nagato, Naohiko Sugita, Yusuke Ito

**Affiliations:** ^1^ Graduate School of Engineering, The University of Tokyo, Tokyo 113-8656, Japan.; ^2^ Innovative Technology Laboratories, AGC Inc., Yokohama 230-0045, Japan.

## Abstract

Femtosecond lasers with extremely high peak intensity have driven remarkable advancements in manufacturing across science, medicine, and industry. However, the problem of notably low machining speed remains unsolved. Here, we demonstrate that by transiently exciting electrons in a transparent material, the laser drilling speed is increased by a factor of 1 million compared to that in multishot percussion drilling. By irradiating with a single shot of a spatially shaped ultrashort laser pulse, the optical properties are momentarily changed on the picosecond scale, making the material considerably easier to machine by a successive laser pulse. The selective absorption of laser energy in regions with excited electrons leads to the rapid heating and evaporation of material at an extraordinarily high speed. Furthermore, the machining is achieved using a low-power light source, four orders of magnitude lower than conventional femtosecond lasers. The concept of transiently altering material properties is expected to usher in a paradigm shift in research and development for manufacturing.

## Teaser

Transient control of optical properties boosts laser machining speed 1 million times via enhanced absorption.

## INTRODUCTION

Transparent materials, such as glass, diamond, and sapphire, have excellent properties such as high hardness, high optical transparency, and exceptional chemical stability, making them indispensable in various industries and societal applications (*1*–*3*). However, the machining of transparent materials has been a challenge for centuries owing to their unique properties. In recent decades, lasers have emerged as a promising solution for machining transparent materials. The development of chirped pulse amplification in 1985 (*4*) has paved the way for the generation of high-power ultrashort pulse lasers (USPLs), with pulse durations ranging from picoseconds (*4*, *5*) to femtosecond (*6*). These USPLs enable the nonlinear absorption of light into transparent materials, facilitating energy transfer for material removal (*7*, *8*). The distinct features of USPL machining have rapidly revolutionized internal and volume processing within transparent materials, including refractive index modification (*9*), nanovoid formation (*10*), elemental redistribution in materials (*11*), and chemical etching rate enhancement (*12*). These innovations have had a notable impact on the development of photonic devices (*13*, *14*), data storage devices (*10*, *15*), three-dimensional (3D) microfluidics, and optofluidics (*12*, *16*). Despite decades of development in high-power laser sources, challenges remain, such as the low speed of material removal owing to weak energy absorption (*17*, *18*) and the generation of damages in transparent materials owing to stress (*19*) and thermal effects (*20*).

To address these challenges, two mainstream strategies—hybrid laser machining (*21*) and tailoring USPL (*22*)—have been explored in recent decades. Hybrid laser machining techniques, such as femtosecond laser–assisted etching (*23*) and water-assisted femtosecond laser machining (*24*), have demonstrated the ability to fabricate microchannels. However, they have limitations in terms of fabrication efficiency (*25*) and structure uniformity in deep drilling (*26*). The second mainstream strategy involves tailoring ultrafast laser pulses, which includes techniques, such as spatially stretching beam shapes, that is, Bessel beams (*27*, *28*), temporally shaping pulses into gigahertz burst pulses (*29*, *30*), temporally combining double or triple pulses (*31*, *32*), and using deep ultraviolet excimer lasers (*33*). The approach has gained substantial attention in recent years owing to its ability to achieve top-down percussion deep drilling of transparent materials with less microcracks, an ultrahigh aspect ratio of up to 1:100 (*29*, *34*), 400-μm-long nanovoids with an aspect ratio exceeding 1:1000 (*35*), and material modifications with an ultrahigh aspect ratio of up to 1:10,000 for stealth dicing of 10-mm-thick glass (*36*). Despite the notable advancement, the time required to create a 1-mm-depth hole using these strategies exceeds 10 s (*29*), posing a formidable challenge for applications in 3D microelectronics and the biomedical industry. These industries require through-glass vias with a fabrication efficiency of up to 1000 holes/s (*37*). Although the possibility of blind nanoholes with lengths ranging from 100 to 400 μm drilled in the nanosecond timescale using single or double Bessel pulses has been demonstrated (*35*, *36*, *38*), generating a through hole remains challenging, and the hole diameter is small, ranging from 100 to 700 nm in these cases. Achieving ultrahigh-speed through-hole drilling in thick (hundreds of micrometers or a millimeter) transparent materials, with aspect ratios greater than 1:100 and time costs notably lower than the millisecond timescale, remains an unmet goal.

Here, we propose to spatially control the transient electronic excitation, which can achieve a fabrication speed of up to 1 million times compared with that of existing percussion machining approaches. We transiently altered the optical properties of transparent materials by generating a long channel of electronic excitation by shaping an ultrashort pulse. This transient channel is immediately ablated by selective heating. This technique can facilitate the drilling of a crack-free through hole with an inner diameter of ~3.1 μm and a depth of 1 mm, resulting in aspect ratios of up to 322. This process can be completed in just 20 μs inside silica glass, with exceptional precision and uniformity.

## RESULTS

### Concept of the proposed method

**Fig. 1.**
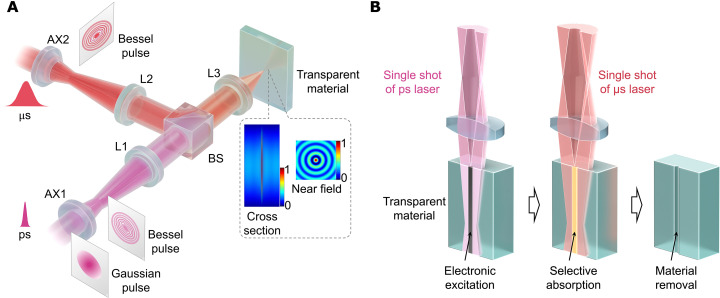
Strategy for ultrahigh-speed machining of transparent materials. (**A**) Schematics of spatial shaping of two pulses. Picosecond and microsecond Gaussian pulses are shaped into Bessel pulses using axicon lenses (AXs) with a base angle of 1°. Two pairs of achromatic lenses (L1 and L2 with a focal length of 400 mm and L3 with a focal length of 30 mm) are used to enhance the intensity of generated Bessel pulses. The illustration of spatial intensity distribution of Bessel pulse along horizontal and vertical directions is presented. BS, beam splitter. (**B**) Schematic of Bessel TSL. A long, transient excited electronic channel (filament) is induced by a picosecond Bessel pulse. The filament is produced uniformly throughout the glass. Because of the increased absorption coefficient at the filament channel, selective and uniform energy absorption of a microsecond Bessel pulse occurs within the entire excited region, heating and removing the materials immediately. Last, a taper-less and crack-free through hole with high aspect ratio is achieved. Note that the microsecond laser pulse was delivered 10 μs before the picosecond laser pulse irradiation in actual experiments.

**Fig. 2.**
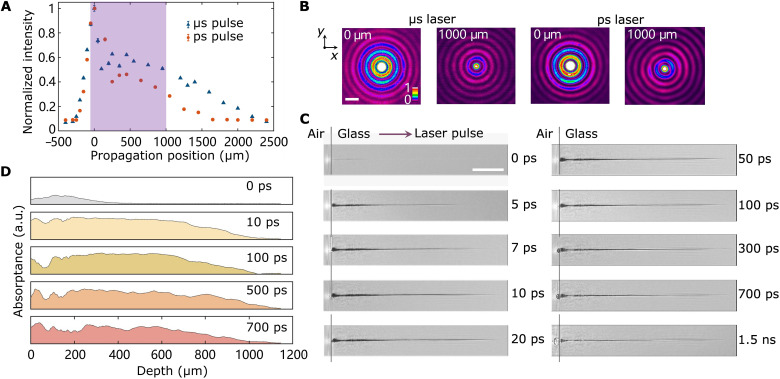
Spatial overlap of Bessel pulses and evolution of induced filament. (**A**) Laser intensity distribution of the center spots of picosecond and microsecond Bessel pulses. Purple rectangle represents the ideal range (−20 to 1 mm) for through-hole drilling used in this study. The focal plane of microsecond laser is defined as the zero position. The error bars indicate the SD. (**B**) Radial intensity distribution of picosecond and microsecond Bessel pulses. Two laser pulses are overlapped (*x* and *y* axes) at a propagation position ranging from 0 to 1000 μm. All images are normalized to their maxima. The full width at half maximum of the microsecond Bessel pulse is ~5.2 μm at the 0-μm position and 4.4 μm at the 1000-μm position, while it is ~4.4 μm at the 0-μm position and 4.2 μm at the 1000-μm position for the picosecond Bessel pulse. Scale bar, 10 μm. (**C**) Filament evolution by a laser pulse duration of 5 ps. The front surface of glass is located at the focal plane of the picosecond laser. Purple arrow indicates the laser incidence. Gray lines denote the interface between the air and the sample. Scale bar, 200 μm. (**D**) Transient absorptance of the probe pulse into filament at different delay intervals. Vertical coordinate ranges from 0 to 1. a.u., arbitrary units.

A long channel of electronic excitation (filament) was generated inside the silica glass by an ultrashort laser pulse formed into a Bessel beam using an axicon lens (*27*). The filament was then heated using a microsecond laser pulse, which was also formed into a Bessel beam using another axicon lens. A schematic of double Bessel pulses—picosecond and microsecond laser Bessel pulses—used in our approach is shown in Fig. 1A. Double Bessel pulses were coaxially aligned and focused on the glass sample for machining (Fig. 2, A and B, and fig. S1A). The intensity features of the generated Bessel pulses are presented as cross-sectional and near-field images. The principle of this approach, referred to as Bessel transient and selective laser (Bessel TSL) absorption, is shown in Fig. 1B. The key feature of this approach is its ability to immediately and uniformly remove the materials throughout the filament using only one shot of picosecond and microsecond Bessel pulses. This allows selective heating for material removal, thus enabling the elimination of stress-induced damage. The selection of the pulse durations of the first and second pulses and the underlying mechanisms for these selections are described in detail and fundamentally discussed in Materials and Methods, Supplementary Text, and figs. S2 to S4. Bessel TSL can simultaneously achieve ultrahigh-speed and high-precision drilling, breaking through the fabrication efficiency limits of existing laser machining techniques.

### Evolution of transient electronic excitation

To investigate the mechanism of ultrahigh-speed drilling, we made phenomena observations using a pump-probe shadowgraph system (see Materials and Methods and fig. S1B) when picosecond laser pulses were focused on glass. The pump pulse was focused onto the sample surface with a pulse energy of 250 μJ, and varying pulse durations of 3, 5, and 10 ps were examined. The interface between the air and the sample was defined as 0 μm in depth. Images were captured with and without pump pulses in each experiment, and the background noise was eliminated through background subtraction. As shown in Fig. 2C and fig. S5, a single continuous filament, induced by the central lobe of the Bessel pulse inside the silica glass, extended along the laser propagation direction from 0 to 10 ps before stabilizing, reaching a length exceeding 1 mm. The filament’s life span within the silica glass was determined to be at least 1.8 ns. In contrast to filaments generated by a Gaussian pulse with a large taper angle (*39*), the filament induced by the Bessel pulse exhibited a uniform black channel over the Bessel zone, a characteristic attributed to the quasi-invariance of Bessel pulse propagation (*40*). The black shadow within the filaments primarily resulted from the probe pulse into the filament (*41*). The transient absorption coefficient at the filament is proportional to and can be evaluated from the absorptance (see Materials and Methods) of the probe pulse into the filament (*42*), as shown in Fig. 2D. Notably, the absorptance remained high at depths up to 1 mm for an extended period from 10 to 500 ps, indicating a filament channel with a high absorption coefficient. In particular, a relatively uniform absorption coefficient was observed within the filament at depths ranging from 200 to 800 μm. Note that the generated filament lastly disappeared, indicating that the material property only changes transiently (see details in Materials and Methods, Supplementary Text, and figs. S6 and S7).

Furthermore, filaments induced by Bessel pulse with a consistent pulse energy of 250 μJ but varying pulse durations were examined (fig. S8). The shorter pulse duration of 3 ps, with potentially higher intensity in the focal region, led to the faster decay of filament channel. To take advantage of the transient electronic excitation, we chose a pulse duration of 5 ps.

### Through-hole fabrication

**Fig. 3.**
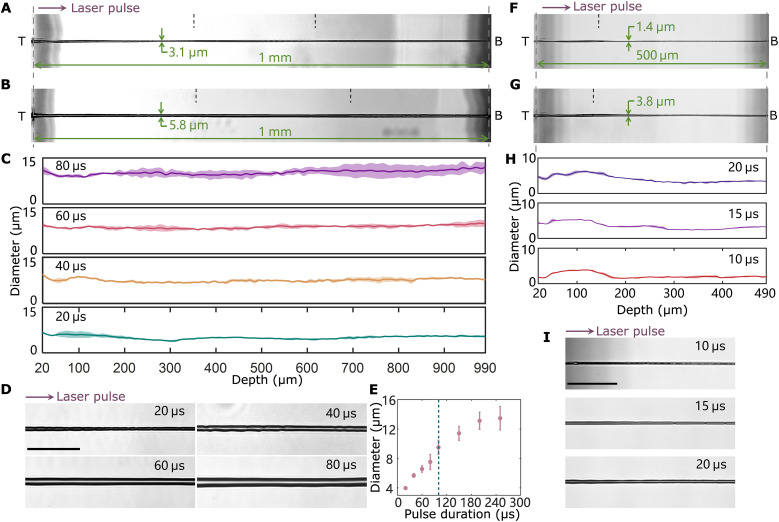
Through holes created by Bessel TSL. Full images of through hole in 1-mm-thick silica glass drilled within (**A**) 20 μs and (**B**) 60 μs. Purple arrow indicates the laser incidence. Green values indicate the diameters at a depth of 300 μm. Three images captured at different depths were assembled to obtain a full image. Black dashed lines indicate the borders of the images. T, top surface; B, bottom surface. (**C**) Diameter variation along the hole depth with different pulse durations of microsecond laser in 1-mm-thick sample. (**D**) Magnified images of 1-mm-depth through hole with different pulse durations of microsecond laser. (**E**) Diameter change with different pulse durations of microsecond laser at a depth of 400 μm for 1-mm-thick sample. Blue dotted line indicates the boundary at a pulse duration of 100 μs, and through holes are not generated when the microsecond laser pulse duration exceeds 100 μs. Full images of through hole in 0.5-mm-thick silica glass drilled within (**F**) 10 μs and (**G**) 20 μs. Black dotted lines indicate the borders of the images. (**H**) Diameter variation along the hole depth with different pulse durations of microsecond laser in 0.5-mm-thick sample. (**I**) Magnified images of 0.5-mm-depth through hole with different pulse durations of microsecond laser. Solid lines in [(C) and (H)] denote average values, and error bars denote the SD. Scale bars, 50 μm [(D) and (I)].

Through-hole images after Bessel TSL drilling of 1-mm-thick silica glass with different pulse durations of microsecond laser, i.e., 20, 40, 60, and 80 μs, are shown, corresponding to pulse energies of 5, 10, 15, and 20 mJ, respectively (Fig. 3, A and B, and fig. S9). The picosecond laser parameters were set as a pulse energy of 250 μJ and a duration of 5 ps for all following experiments unless otherwise stated. Note that the microsecond laser pulse was delivered 10 μs before the picosecond laser pulse, and the microsecond laser pulse energy is defined as the energy delivery after the picosecond pulse irradiation in this study. Our results demonstrated that a taper-less through hole with a depth of 1 mm and an inner diameter of ~3.1 μm, corresponding to an extremely high aspect ratio of ~322, all achieved within a mere 20 μs. The ultrafast drilling speed achieved by our method is ~1 million times faster than that in recent percussion drilling studies (*29*, *33*). When the pulse duration is extended to 60 μs, the diameter of the through hole increases to 5.8 μm. The magnified images in the middle area of the through hole, with different pulse durations of microsecond laser (Fig. 3D), reveal a high level of uniformity in hole shape with a glossy wall surface, free of microcracks and chipping. A quantitative comparison of diameter along the hole depth with different pulse durations of microsecond laser was performed to identify the uniformity and controlling capability of hole shape in Bessel TSL (Fig. 3, C and E). The measurements were based on microscope images of the through-hole cross sections (see Materials and Methods). A wider through hole can be produced by increasing the pulse duration of microsecond laser when the pulse duration does not exceed 100 μs. The hole diameter expands at a rate of ~1.2 μm/20 μs, indicating that the shape of drilled hole can be controlled to a certain extent. There is saturation in the diameter of the drilled through hole, i.e., ~13.5 μm, for 1-mm-thick silica glass sample using the current pulse energies of the picosecond laser and microsecond laser (see Supplementary Text and fig. S10). The larger hole diameter is attributed to the continuous heating of vaporized materials, resulting in an extended affected zone of energy deposition and a larger volume of vaporized materials.

Notably, the diameter of the hole drilled using this method was smaller than the spot size of the central lobe of microsecond Bessel pulse, which is ~5.2 μm at half maximum. This result also indicates that the microsecond laser pulse was selectively absorbed into the filament. Typically, high-power continuous wave lasers have difficulty machining small structures; however, our method showcases the potential for micromachining without being constrained by the feature size limitations of high-power lasers.

We conducted the same experiments for 0.5-mm-thick silica glass drilling (Fig. 3, F and G, and fig. S11). A though hole with the highest aspect ratio of up to 384, 500 μm in depth, and 1.3 μm in inner diameter can be achieved within 10 μs. Compared with the 1-mm-thick sample, the through hole can be drilled with a shorter pulse duration of the microsecond laser, indicating that less energy is required for thin glass. The holes created in both 0.5- and 1-mm-thick glass samples using the same pulse duration of the microsecond laser exhibited similar diameters and shapes at depths greater than 200 μm (Fig. 3, C, H, and I; and fig. S11C), showcasing the precision of diameter control in this method. The saturation in the diameter of the drilled through hole, i.e., ~15.5 μm, for 0.5-mm-thick silica glass sample was also observed (see Supplementary Text and fig. S11). A key advantage of Bessel TSL is its ability to achieve through-hole drilling within tens of microseconds, regardless of sample thickness, provided that the filament is long ranged.

### Material removal dynamics

**Fig. 4.**
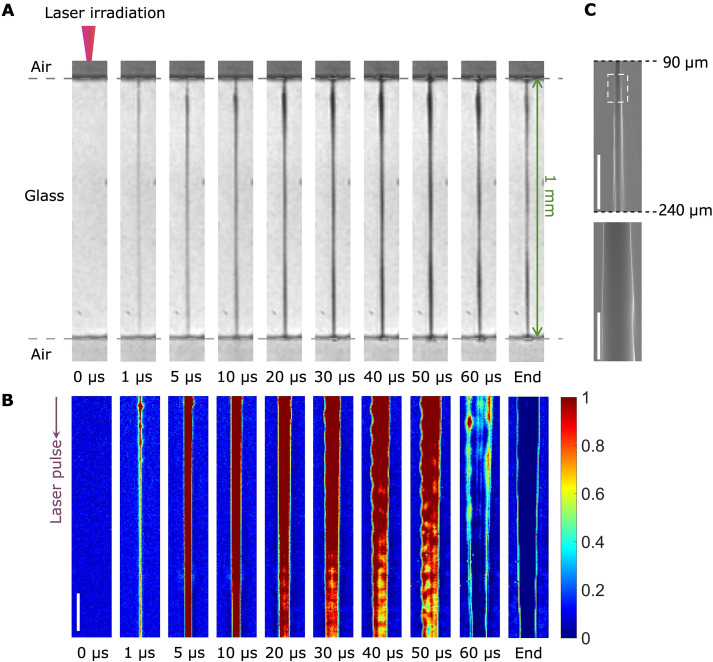
Observation of through holes. (**A** and **B**) Time-resolved sequence of through-hole drilling using Bessel TSL, with the evolution monitored over a time domain ranging from 0 to 60 μs. Images in (B) are the magnified area in the middle of the drilling region in (A). Colors in (B) represent the normalized brightness of the luminance. Red color means the brightest region, while blue color means the darkest region. Scale bar, 10 μm. Two Bessel pulses arrived from the top, with dotted gray lines referring to the interface between the air and the glass. (**C**) SEM images of the cross section of the 1-mm-depth hole after polishing. The first image was captured at a depth ranging from 90 to 240 μm. The second image indicates the magnified area within the dotted white rectangle in the first image. Scale bars, 50 μm (first image) and 10 μm (second image).

The ultrafast drilling phenomena of a 1-mm-depth through hole in Bessel TSL using a pulse duration of 60 μs and a pulse energy of 15 mJ were investigated (Fig. 4, A and B, and movie S1). Initially, no material removal occurred at 0 ps despite the delivery of a microsecond laser 10 μs prior, attributed to the large bandgap and low absorption coefficient of silica glass. However, upon delivery of a picosecond Bessel pulse, a transiently elongated filament exceeding 1 mm in length was generated inside the sample (Fig. 2C). This filament, primarily composed of excited electrons, exhibited high localized absorptivity owing to semimetallization (*38*), leading to abruptly improved energy absorption of the microsecond laser pulse. The microsecond Bessel pulse can achieve uniform energy deposition over the generated filament channel (*35*), resulting in heating and material removal, ultimately producing a through hole within 10 μs. This is evidenced by the fully distributed black region in Fig. 4A and the magnified bright region in Fig. 4B. As the irradiation time of the microsecond laser increased from 10 to 50 μs, the removed materials continued to be ejected from the top and bottom surface (figs. S12 and S13), causing the hole shape to gradually shift toward the radial direction, with the inner diameter increasing from ~5.6 to 9.3 μm. The final hole shape, with an inner diameter of 6.6 μm, can be clearly seen at ~40 μs after the irradiation ceased. Luminescence was observed during machining (Figs. 4B and fig. S12). Because of its close association with drilling processes (*43*, *44*), luminescence can be used to quickly evaluate the quality when machining thousands of holes. The dedicated drilling setup is shown in Materials and Methods and fig. S14. In situ evaluation was successfully achieved (figs. S15 and S16).

To verify that the drilled hole penetrates through the 1-mm-thick sample, we conducted scanning electron microscopy (SEM) observations of the cross section of the drilled hole (Fig. 4C). Because of the small size and large aspect ratio (over 100) of the drilled hole, only a part of the cross section could be observed after polishing. In addition, ink was penetrated into the drilled holes (see Materials and Methods and fig. S17). The through hole was filled with yellow ink, proving that no plugging by debris or resolidified materials exists inside and at the entrance of the drilled hole. The SEM images and ink images provide evidence of a smooth inner wall of the through hole, indicating its potential for various industrial applications, such as biofilters and 3D interposers.

### Applicability to diverse materials

**Fig. 5.**
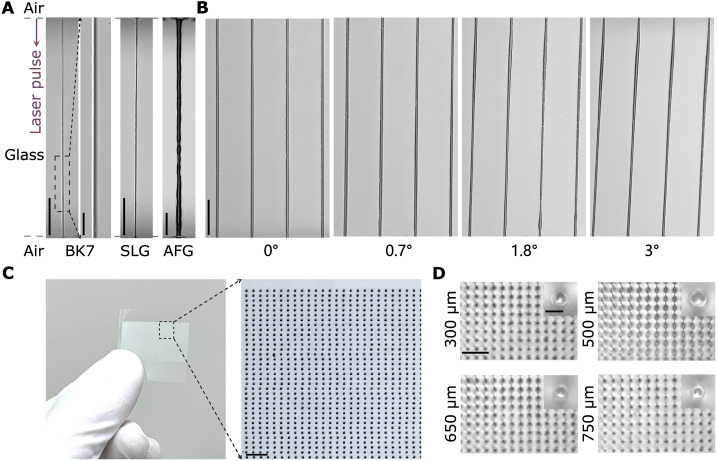
Fabrication of different features. (**A**) Microscope images of through holes in different glass materials drilled within 60 μs, i.e., BK7, AFG, and SLG. The second image of BK7 indicates the magnified area within the dotted black rectangle in the first image of BK7. Scale bars, 10 μm (second image) and 50 μm [other images in (A)]. Two Bessel pulses arrived from the top, with dotted gray lines referring to the interface between the air and the glass. (**B**) Microscope images of tilted holes in 1-mm-thick silica glass drilled within 60 μs. Scale bar, 100 μm. (**C**) Thousands of through holes fabricated in 1-mm-thick silica glass. Scale bar, 300 μm. (**D**) Transverse microscopy images of hole arrays with different depths. Scale bars, 300 and 10 μm (magnified image).

The ultrahigh-speed machining capability of this method in other transparent materials is verified. The through-hole fabrication is successfully achieved in widely used glass materials, i.e., borosilicate crown glass (BK7), soda lime glass (SLG), and alkali-free glass (AFG; AGC Inc.), within tens of microseconds, as shown in Fig. 5A, figs. S18 to S20, and movies S2 to S4. The ultrafast dynamics of material removal shown in figs. S21 and S22 demonstrate the universality of this method in sapphire and silicon carbide (SiC) materials (see Supplementary Text), which are crucial materials for application in the semiconductor field (*1*). Tilted hole fabrication using Bessel TSL is also achieved, as shown in Fig. 5B and fig. S23, which shows great potential in photonics and electronics applications (*45*).

Thousands of through holes are fabricated in 1-mm-thick silica glass using a drilling strategy involving a fixed laser spot and continuously moving glass sample (see Materials and Methods, Fig. 5C, and movie S5). The minimum pitch between holes for stable hole array fabrication is 80 μm for 1-mm-thick and 50 μm for 0.5-mm-thick silica glass samples, respectively. We note that because of the ultrahigh-speed drilling for each hole in our method, the fabrication speed is dependent on the stage velocity. For example, achieving 1000 holes per line within 1 s or less is easily attainable by setting the repetition rate of two Bessel pulses at 1 kHz or higher. Unlike existing laser machining techniques, our method eliminates the need for chemical etching or other postprocessing steps. Using identical conditions, transverse microscopy images of holes with depths of 300, 500, 650, and 750 μm demonstrate the high repeatability and stability of our proposed method, with uniformity in size and shape (Fig. 5D). By further spatially controlling the Bessel core regions of picosecond and microsecond pulses, the uniformity of the fabricated thousands of through holes, such as the variance of hole diameters, can be improved (see details in Supplementary Text and fig. S24).

## DISCUSSION

By assigning different roles to the two laser pulses, we achieved ultrahigh-speed laser machining of transparent materials at a speed 1 million times faster than that of conventional percussion machining methods. Traditionally, notable effort has been put into developing high-intensity lasers that store high energy in short pulse durations (around 10^−13^ s). In contrast, we found that extending the pulse duration to the microsecond range, a completely opposite approach, makes machining transparent materials remarkably easier. With conventional high light intensity, plasma reflection occurs, preventing efficient energy delivery (*46*). In our study, we used a microsecond laser pulse with a light intensity on the order of 100 W, which is four orders of magnitude lower than that of conventional methods. By suppressing plasma reflection, remarkably high-efficiency machining was achieved.

This research contributes to fostering basic scientific research and advancing industrial technologies: High aspect ratio holes are essential for high-sensitivity nuclear magnetic resonance measurements, allowing for the elucidation of material properties under high pressure in diamond anvil cells (*47*); the semiconductor industry requires through holes in glass and SiC substrates (*48*, *49*); and glass biofilters are required for rapid diagnostics (*50*). Furthermore, this research signifies a paradigm shift in material machining, where changing the material properties transiently can alter the physics of machining and markedly improve the results.

## MATERIALS AND METHODS

### Bessel TSL experiment

#### Optical setup

Figure S1A shows the detailed optical setup used in this study. A picosecond laser (Pharos SP 10W, Light Conversion) with a central wavelength of 1030 nm and a pulse duration of 5 ps was used, with a pulse energy of 250 μJ for machining. In addition, a microsecond laser with a wavelength of 1070 nm emitted from a fiber laser oscillator (redPOWER R4 500W, SPI Lasers) was used. Both pulses were converted from a Gaussian to a Bessel pulse through the axicon lenses AX1 and AX2, with the same base angle of 1° (AX251-B, Thorlabs). After passing through achromatic lenses L1 and L2, with a focal length of 400 mm (AC254-400-B, Thorlabs), two Bessel pulses were merged coaxially using a beam splitter (BS014, Thorlabs) and focused onto the sample by an achromatic lens L3, with a focal length of 30 mm (AC254-030-B, Thorlabs). For illumination, light produced by a laser lamp (CAVILUX HF, Cavitar), with a wavelength of 646 nm and a laser fluence of 3.98 × 10^−7^ J/cm^2^, was directed through the sample from a direction perpendicular to the optical axis of the laser pulses for machining, which were collected using an objective lens L6 with a numerical aperture of 0.4 (M Plan Apo NIR 20×, Mitutoyo). Images were obtained using a tube lens L7 (TTL200, Thorlabs) with a focal length of 200 mm and observed using a high-speed camera at a speed of 1 million frames/s and an exposure time of 200 ns (HPV-X2, Shimadzu). To minimize the disturbance caused by luminescence during machining and enhance observation accuracy, a band-pass filter (BPF) was applied, which was removed when observing the luminescence generated. The irradiation timings of two Bessel pulses were set using a digital delay generator (DG645, Stanford Research System). The microsecond laser pulse was delivered 10 μs before the picosecond laser pulse irradiation.

#### Spatial overlap of two Bessel pulses

For the measurement of the laser intensity along the propagation direction (Fig. 2A) and the radial overlap of two Bessel pulses (Fig. 2B), the sample was removed. The setup within the blue dashed rectangle in fig. S1 was used. After passing through an objective lens L4 (M Plan Apo NIR 50×, Mitutoyo) and a tube lens L5 (TL-Y3, SIGMAKOKI CO. LTD.), the generated Bessel pulses were magnified and captured using a beam profiler (SP620U, Spiricon). Subsequently, the spot size, laser intensity, and radial overlap of generated Bessel pulses along the propagation direction were measured by adjusting the position of L4. The measured focal plane of picosecond and microsecond Bessel pulses is shown in the top left panel of fig. S1A, revealing only a 10-μm vertical shift between the two focal planes.

### Pump-probe experiment

#### Optical setup

The experimental setup of pump-probe is shown in fig. S1B. The same picosecond laser was used to generate picosecond pulses, with a wavelength of 1030 nm, a pulse energy of 250 μJ, and different pulse durations of 3, 5, and 10 ps. Each delivered pulse was divided into pump and probe pulses using a beam splitter. An axicon lens converted the pump pulse into a Bessel pulse, which was then focused onto a sample using two achromatic lenses L1 (AC254-400-B, Thorlabs) and L2 (AC254-030-B, Thorlabs). The probe pulse was frequency doubled using a beta barium borate crystal and then illuminated the sample perpendicularly to the pump pulse. A short-pass filter (SPF; FES0950, Thorlabs) was used to eliminate the remaining 1030-nm light from the probe pulse. The transmitted probe pulse was captured using an apochromatic objective lens L3 (M Plan Apo NIR 20×, Mitutoyo) and a tube lens L4 (TTL200, Thorlabs). The filament shadowgraph was imaged on a cooled charge-coupled device (CCD; BU-55LN, Bitran). The CCD was synchronized with the picosecond laser system to ensure that each image corresponded to a pump pulse. Time-resolved imaging was realized using an optical delay line comprising two reflectors, allowing for probe pulse delays ranging from femtoseconds to nanoseconds. The zero-delay position was determined by aligning the optical path lengths of the pump and probe pulses. This reference zero-delay position was established in silica glass when the filament was weak and short (Fig. 2C). Images with increasing probe delays were recorded sequentially, which revealed the evolution of the filaments. A BPF was applied to minimize the disturbance from the pump pulse and the emissions during filament. Another CCD was used to locate the surface of the sample under white-light illumination.

The focal position of the pump pulse within the samples was determined. Initially, the sample surface was identified using a white-light CCD. Subsequently, a single pump pulse was used to drill a piece of silica glass sample, creating numerous holes by moving the sample along the beam propagation direction with a step size of 10 μm. The focus was identified as the sample position where the smallest hole was produced. The sample was fixed on a three-directional moving stage, allowing for adjustments of the incidence points and the focal positions of the pump pulses on the sample.

#### Absorptance of the probe pulse into filament

First, images with and without pump pulse were recorded in each experiment with different time delays. By calculating the ratio between these images, we eliminated background noise. Using ImageJ software, we measured the gray values of the full images after dividing them. The gray values along the central line of the filament, normalized to their maximum, were used to assess the absorptance of the probe pulse. A higher gray value, indicating a darker color in the images, corresponded to a higher absorptance.

Because of the cylindrical symmetry of the transient electronic excitation within the filament along the pump pulse, the probe pulse experienced absorption, reflection, and refraction, as it passed through the filament channel. In our study, we focused on the central part of the filament that aligns with the axes, allowing us to ignore refraction. For simplicity, the reflection was also neglected, and only the absorption was considered. The transient average absorption coefficient of filament can be calculated (*42*). Thus, the transient absorption coefficient at the filament channel is proportional to and can be evaluated using the absorptance of the probe pulse into the filament.

### Calculation of the electron density inside the filament induced by the first pulse

#### Calculation based on experimental results

To further confirm that no internal modification occurred inside the silica glass after the delivery of a picosecond Bessel pulse with a pulse energy of 250 μJ, the electron density inside the filament was calculated for internal modification evaluation. The maximum probe absorbance at each position along the depth direction was extracted from filament images in the experiment. Subsequently, the electron density in the center of the filament was determined according to the Drude model (*17*). The detailed calculation is shown in Supplementary Text.

#### Calculation based on simulation

To explore the effect of the pulse duration of the first pulse on the deposited energy used for filament formation, we calculated the electron density variation when using different laser pulse durations. We considered a single point on the sample surface irradiated by an ultrafast pulse with different pulse durations. According to the rate equation (*51*) and Fresnel equation, total reflection will occur when the electron density reaches the critical value, i.e., 1.05 × 10^27^ m^−3^ (*52*). Therefore, we assume that the electron density cannot exceed this critical value. In the simulation, the pulse energy is 250 μJ, and the laser pulse diameter is 4.4 μm on the sample surface. The detailed calculation is provided in Supplementary Text.

### Calculation of the energy absorption dependence on the second laser pulse durations

When the pulse duration of the second laser pulse differs, the absorbed energy varies even if the pulse energy is the same because of the effects of heat conduction and electron density–dependent Fresnel reflection. We estimated the dependence of absorbed energy on the pulse duration through the calculation of light absorption into the filament formed by the first laser pulse. First, the electron density distribution in the filament formed by the first laser pulse was estimated from the image obtained by the pump-probe experiment. Then, the propagation of the second laser pulse inside the material was calculated by considering the electron density–dependent absorption and reflection and heat conduction. The absorbed energy was determined by integrating the absorbed energy density over space and subsequently integrating it over time. The results showed that the amount of absorbed energy reached its maximum when the pulse duration was 30 μs. The detailed calculation model is shown in Supplementary Text and table S1.

### Timing control of the picosecond and microsecond pulses

To achieve the precise timing control of the two pulses, we first detect the timing difference of two laser pulses using two photo detectors (818-BB-21, Newport) connected to a high-frequency oscilloscope with nanosecond-scale resolution. Subsequently, the irradiation timing of the two lasers is adjusted using this delay generator with a resolution of 1 ns. Note that we have experimentally confirmed that there is no fluctuation during the timing control. By doing so, we can precisely set the delivery timing of these two pulses with nanosecond-scale resolution, which is much smaller than the actual timing delay (10 μs) and drilling time (microsecond timescale) in our experiments.

### Hole diameter extraction

Three images were captured at the top, middle, and bottom of each hole using a laser microscope (LEXT OLS4100, Olympus). The brightness and contrast of the images were adjusted to ensure consistency. Subsequently, full cross-sectional images of each through hole were created by assembling two or three images, and the hole diameters along the depth were extracted from the hole edges using Python. All values were measured in three holes drilled using the same laser parameters, and the average values and SDs were calculated.

As shown in fig. S11, for pulse durations of 40 μs or longer, inconsistencies in hole diameter were observed at depths shallower than 200 μm. This can be attributed to the nonuniform energy absorption into filament induced by the intensity inhomogeneities of the microsecond Bessel pulse (Fig. 2A), nonuniform distributed filament near the sample surface (Fig. 2C and fig. S5), and the unstable Bessel pulse propagation regime near the sample surface (*53*). As shown in figs. S9 to S11, the thickness dependence of the uniformity may originate from the size of the plume generated near the top surface. A larger plume leads to stronger light shielding near the top, resulting in greater nonuniformity of light intensity inside the glass.

### Fabrication of tilted through holes

The silica glass sample was fixed on a rotation stage (GN05, Thorlabs), and the sample was rotated for a specific angle before the tilted through-hole fabrication experiments. Note that the direction of laser propagation is fixed for all experiments.

### Fabrication of thousands of through holes

The Bessel pulses were focused on a glass sample that was fixed on a specially designed fixture and a high-speed stage (MLS203, Thorlabs), as shown in fig. S14A. During the fabrication of thousands of through holes, a drilling strategy involving a fixed laser spot and continuously moving glass sample was applied, as shown in fig. S14B. The high-speed stage moved at a velocity of 100 mm/s, with a pitch of 100 μm along the *x* and *y* axes. The pulse duration of the microsecond laser was 60 μs, and the repetition rate of picosecond and microsecond laser pulses was set to 1 kHz, resulting in a fabrication speed of 1000 holes/s. A coaxial high-speed monitoring method using a photodetector was developed to collect luminescence during the drilling of thousands of holes. The luminescence was passed through a glass sample and collimated using two objective lenses with a focal length of 75 mm (AC254-075-A, Thorlabs). A notch filter and SPF (SHPF-950, Optosigma) were applied to decrease the disturbance caused by incident laser pulses. Subsequently, the luminescence was measured using a photodetector (R10467U-50-01, Hamamatsu), and the optical signal of the plasma light was recorded using a data acquisition device (USB-6336, National Instruments) with a sampling frequency of 1 MHz. The number of rows and columns of holes, pitch between two adjacent holes, and total fabrication time were controlled by the high-speed stage. After drilling the first line of through glass vias along the *y* axis, the glass sample was moved for a pitch distance along the *x* direction for second-line drilling.

### High-speed evaluation of hole quality based on luminescence

Luminescence was not blocked during machining (fig. S12) to investigate the energy absorption process of the microsecond laser using a high-speed camera (HPV-X2, Shimadzu). The bright area can be observed from the luminescence originating from the filled vaporized materials, indicating simultaneous heating of the entire material region within microseconds.

As is evident from the luminescence (fig. S12) and drilling phenomena (figs. S12 and S13), the photodetector captures real-time luminescence signals at a sampling rate of 1 MHz to achieve an instant evaluation of the hole type, that is, through and blind holes. The intense absorption of the microsecond laser in blind hole drilling results in prolonged emission of strong luminescence in nonpenetrated regions. This results in a higher amplitude and longer duration of optical signal compared with through-hole drilling (figs. S15 and S16). The distinct optical signals offer a valuable means of evaluating drilling performance, even during ultrahigh-speed machining such as Bessel TSL drilling. Figure S14D shows the signal profiles of 500 holes drilled with pulse durations of 60 μs. The features of signal profiles demonstrate that all the drilled holes are through holes. Moreover, to assess the quality of each hole in a large-scale drilling operation, a one-to-one mapping of optical signals and hole positions [defined by coordinates (*m*, *n*)] has been established (figs. S14E and S16). The developed optical monitoring system is valuable for the high-speed evaluation of machining performance during ultrahigh-speed laser machining without time-consuming camera observations.

### Materials and structure characterization

Synthetic silica glass with dimensions of 75 mm by 25 mm by 1 mm and 75 mm by 25 mm by 0.5 mm (AQ series, AGC Inc.) was used as the sample material. The surfaces of the samples were polished to achieve a surface roughness of less than 10 nm. The glass sample with drilled through holes underwent further polishing using a cross-sectional polisher. The morphologies of the cross sections of the drilled through holes were then analyzed using SEM (JSM-6510LA). Optical images of the drilled holes were acquired using a laser microscope (LEXT OLS4100, Olympus) after cleaning the samples for 5 min using an ultrasonic cleaning machine to eliminate any unwanted deposition.

N-BK7 (Schott) and SLG (AS2, AGC Inc.) with dimensions of 75 mm by 25 mm by 0.3 mm and AFG (AN100, AGC Inc.) with dimensions of 75 mm by 25 mm by 0.5 mm were used in the material independence experiments. Sapphire (Al_2_O_3_, Crystal Base Co. Ltd.) with dimensions of 75 mm by 25 mm by 1 mm and SiC (4H-SiC, IV-SemiteC) with dimensions of 75 mm by 25 mm by 0.5 mm were used. The surfaces of the above samples were polished to achieve a surface roughness of less than 10 nm. Microscopic observation of the drilled holes was performed using the same cleaning strategy as silica glass experiments for all the above materials.

#### Ink experiments

A silica glass sample with drilled through holes was submerged in an ink solution (G40-5700, Monotaro) within a beaker, waiting for 30 min to allow the ink to completely flow into the through holes. Subsequently, two glass substrates of the same sample size as the sample were affixed to both sides of the sample to prevent ink leakage from the holes. Last, microscopic observations of the through holes filled with ink were performed.

## Supplementary Materials

### The PDF file includes:

Supplementary Text Figs. S1 to S24 Table S1 Legends for movies S1 to S5 References
