# Supplementary material for: Ultrahigh-speed laser drilling of transparent materials via transient electronic excitation
Source: Sci Adv. 2025 Jun 11;11(24):eadv4436. doi: 10.1126/sciadv.adv4436 (PMC12153975; doi:10.1126/sciadv.adv4436)
Supplement: Supplementary file 1 — Supplementary Text Figs. S1 to S24 Table S1 Legends for movies S1 to S5 References [file sciadv.adv4436_sm.pdf]

Science Advances
AAAS

Supplementary Materials for

Ultrahigh-speed laser drilling of transparent materials via transient electronic excitation

Yanming Zhang *et al.*

Corresponding author: Yusuke Ito, y.ito@mfg.t.u-tokyo.ac.jp

Sci. Adv. **11**, eadv4436 (2025)
DOI: 10.1126/sciadv.adv4436

The PDF file includes:

Supplementary Text
Figs. S1 to S24
Table S1
Legends for movies S1 to S5
References

Other Supplementary Material for this manuscript includes the following:

Movies S1 to S5

Supplementary Text

Selection of the pulse durations of the first and second pulses

Selection of the pulse duration of the first pulse

As described in the section of "Concept of the proposed method", a long channel of electronic excitation (filament) needs to be firstly generated inside the transparent materials by an ultrashort laser pulse, to achieve Bessel TSL. The lifetime and intensity (related to the transient absorption coefficient for the second pulse) of the generated filament, are affected by the pulse duration of the first pulse. They determine the energy absorption of the second pulse and the final processing quality.

Firstly, the lifetime of the filament, induced by a picosecond pulse inside silica glass, is 100 times longer than that induced by a femtosecond pulse, as previously reported (*54*), even though they used a Gaussian beam. A long lifetime of filament is essential for sufficient energy absorption of the second pulse. Secondly, the energy absorption used for filament formation in a ps pulse is much higher than that in a femtosecond (fs) pulse. The variation of excited electron density is used to evaluate the energy absorption of the first pulse. To calculate the excited electron density, the rate equation is used (*51*):

$$\frac{\partial \rho}{\partial t} = \sum_k \sigma_k I^k + \sigma_c I \rho - \eta_{rec}\rho^2 - \eta_{diff}\rho \qquad \text{(S1)}$$

where ρ is the density of excited electrons, t is the time, I is the pump pulse intensity, σ_c is the cascade ionization coefficient, η_{rec} and η_{diff} are the electron recombination and diffusion coefficients, respectively, k indicates k-photon absorption, σ_k is the k-photon ionization coefficient. Based on the Fresnel equation, total reflection will occur when the electron density reaches the critical value. Therefore, we assume that the electron density cannot exceed the critical value. As shown in fig. S2, when the same pulse energy of 250 μJ and different pulse durations of 300 fs, 3 ps and 5 ps are applied, the generation of a high-electron-density surface (reaching critical electron density), induced by an fs pulse, is much faster than that by a ps pulse, leading to less energy deposition used for filament formation. It can be observed from fig. S2 that the laser energy absorption, used for electron excitation, in 3 ps and 5 ps pulses is 8 and 11 times higher than that in a 300-fs pulse, respectively. This is primarily attributed to the strong photoionization caused by the high peak intensity of the fs pulse. Note that the value of the reaching time in fig. S2 is only used to compare the energy absorption ratio with different pulse durations of the first pulse, and it may not be precise to evaluate the actual absorption time. Once the electron density reaches the critical value, subsequent laser energy of this pulse is either absorbed at that surface or reflected from it (*51*), suppressing the laser energy deposition for filament formation. In contrast, when the pulse duration is in the order of picoseconds, the electron density does not increase rapidly, allowing for more efficient laser energy deposition along the central core of Bessel beams compared to fs pulses. This observation is consistent with previous research (*55*, *56*). Thus, to produce a long and uniform filament with high electron density, a pulse duration of a few picoseconds is selected. Moreover, if the pulse duration is further increased to tens or hundreds of picoseconds or longer, efficient absorption does not occur because of the weak nonlinear absorption.

The experiments were conducted to verify the dependence of pulse duration and pulse energy of the first pulse on drilling quality in Bessel TSL processing of silica glass. A constant pulse

duration of 100 μs and pulse energy of 25 mJ for μs pulse were used. As shown in fig. S3, a through-hole can be successfully achieved with a pulse duration larger than 3 ps and a pulse energy larger than 200 μJ. Otherwise, unsuccessful through-holes, such as blind holes or no holes, were generated. Thus, based on the simulation and experimental results, the pulse duration of the first pulse is selected in the range of 3 ps to 10 ps for stable through hole fabrication.

Selection of the pulse duration of the second laser pulse

As described in the section of "Concept of the proposed method", The key feature of the approach is its ability to immediately and uniformly remove the materials throughout the filament, which is mainly affected by the second pulse. Both of the laser intensity and real deposited energy absorbed for material removal are critical to successful achieve through hole fabrication in Bessel TSL.

If the pulse duration of the second pulse is in the range of fs or ps timescale, small material removal can be observed when the low pulse energy (from few μJ to tens of μJ) is applied, as reported before (*38*), but only blind nano-holes can be generated using double Bessel pulse. Through hole is difficult to be generated and the diameter of the hole is small (ranging from 100 to 700 nm). If high pulse energy is applied, as mentioned above, the fast generation of high-electron-density surface (reaching critical electron density), because of the extremely high laser peak intensity, will limit the energy deposition for material removal (*57*, *58*).

As shown in our simulation (detailed method is shown below) results fig. S4, if the pulse duration of the second laser pulse is smaller than 1 μs, the energy absorption of the second laser pulse is low because of the large reflection. Note that the same pulse energy of 15 mJ was applied for all conditions. If the pulse duration is between 1 μs (15 kW) and 70 μs (214 W), the energy absorption is large and reaches the largest value when the pulse duration is 30 μs (500 W). If the pulse duration is larger than 100 μs (150 W), the energy absorption rapidly drops due to thermal diffusion. Under the same pulse energy, if we use continuous-wave laser, i.e. longer pulse duration, blind holes or even no holes occur.

Based on the above results, the pulse duration of the second laser pulse is selected in the range of 20 μs to 80 μs for sufficient material removal.

<u>Simulation of energy absorption dependence on the second laser pulse durations</u>

Overview

When the pulse duration of the second laser pulse differs, the absorbed energy varies even if the pulse energy is the same, because of the effects of heat conduction and electron-density-dependent Fresnel reflection. Here, we estimate the absorbed energy when a second laser pulse with different pulse durations (but the same pulse energy) is delivered to the filament formed by the first laser pulse. Through this analysis, we evaluate the dependence of absorbed energy on the pulse duration of the second laser pulse. The physical parameters used in the simulation is displayed in Table S1.

Electric Field and Intensity Distribution of the Second Laser Pulse

The electron density distribution in the filament formed by the first laser pulse was estimated from the image obtained by the pump-probe experiment. The distribution was estimated using the Drude model and the inverse Abel transformation (*59*).

We model the situation in which the second laser pulse is delivered to the filament. Because the second laser pulse propagates while being absorbed and reflected by the filament, the conventional expression for the spatial distribution of a Bessel beam cannot be directly applied. Therefore, we first define the electric field distribution at the sample surface and then consider the light propagation in the *z*-direction (perpendicular to the surface, into the material). When a Gaussian beam passes through an axicon lens and two achromatic lenses, the electric field distribution at the sample surface $E_s(r)$ is given by the following equation:

$$E_s(r) = E_0 \exp\left(-\frac{r^2}{r_0^2}\right)\exp(ikr\sin\theta), \tag{S2}$$

where E_0 is the electric field amplitude at the center, r is the radial distance from the center, r_0 is the beam radius at the sample surface, k is the wavenumber, and θ is the inclination angle of the wavefront relative to the sample surface. In this analysis, θ is set to 3.4°. The electric field distribution inside the material, $E(r,z)$, is then numerically obtained as the light propagate inside. Subsequently, the time-averaged light intensity distribution, $I(r,z)$, is obtained using the following equation:

$$I(r,z) = \frac{1}{2} n_0 c_0 \varepsilon_0 |E(r,z)|^2, \tag{S3}$$

where n_0 is the refractive index of the glass medium, c_0 is the speed of light in free space, and ε_0 is the permittivity of free space.

Temperature Dependence of the Absorption Coefficient

The relationship between temperature and the absorption coefficient is given by the following equation (*60, 61*):

$$\alpha(T) = \frac{3}{2} U_{\mathrm{gap}} \alpha_c \rho(T), \tag{S4}$$

where U_{gap} is the bandgap energy of the glass, and $\rho(T)$ is the temperature-dependent electron density. The term α_c is expressed as

$$\alpha_c = \frac{1}{\omega^2\tau^2 + 1} \frac{e^2\tau}{n_0 c_0 \varepsilon_0 m_e \frac{3}{2} U_{\mathrm{gap}}}, \tag{S5}$$

where e is the elementary charge, ω is the angular frequency of the laser light, τ is the relaxation time of free electrons, m_e is the electron mass.

The electron density $\rho(T)$ is given by

$$\rho(T) = \rho_b \frac{3\sqrt{\frac{\pi}{2}}\left(\frac{k_B T}{U_{\mathrm{gap}}}\right)^{\frac{3}{2}} \exp\left(-\frac{U_{\mathrm{gap}}}{2k_B T}\right)}{1 + 3\sqrt{\frac{\pi}{2}}\left(\frac{k_B T}{U_{\mathrm{gap}}}\right)^{\frac{3}{2}} \exp\left(-\frac{U_{\mathrm{gap}}}{2k_B T}\right)}, \tag{S6}$$

where ρ_b is the bound electron density, and k_B is the Boltzmann constant.

Fresnel Reflection

According to Fresnel's equations, the amplitude reflection coefficients for s-polarized and p-polarized light, denoted as r_s and r_p, are given by

$$r_s = \frac{n_1\cos\theta_1 - n_2\cos\theta_2}{n_1\cos\theta_1 + n_2\cos\theta_2} \tag{S7}$$

$$r_p = \frac{n_2\cos\theta_1 - n_1\cos\theta_2}{n_2\cos\theta_1 + n_1\cos\theta_2}, \tag{S8}$$

where n_1 and n_2 represent the refractive indices of the incident and transmitted media, respectively, and θ_1 and θ_2 denote the angles of incidence and refraction.
Considering the random polarization of the laser, we assumed that the reflectance R is the average of the intensity reflectance for both polarizations:

$$R = \frac{r_s^2 + r_p^2}{2}. \tag{S9}$$

The refractive index with excited electrons $n(\rho)$ can be expressed as

$$n(\rho) = n_0 \sqrt{1 - \frac{\rho e^2}{n_0^2 \varepsilon_0 m_e \omega^2}} \tag{S10}$$

This indicates that as the electron density increases, the refractive index decreases, resulting in the variation of the reflectance. Especially, when the electron density reaches a certain value, total reflection occurs.

Heat Conduction
The temporal change of the temperature distribution was calculated using the heat conduction equation in cylindrical coordinates:

$$\rho_{\mathrm{mass}} C_{\mathrm{v}} \frac{\partial T}{\partial t} = \kappa \left(\frac{1}{r}\frac{\partial T}{\partial r} + \frac{\partial^2 T}{\partial r^2} + \frac{\partial^2 T}{\partial z^2} \right) + Q, \tag{S11}$$

where ρ_{mass} is the mass density, C_{v} is the specific heat, κ is the thermal conductivity. The absorption of the second laser pulse energy is expressed as Q. The implicit finite difference method was used to calculate the heat conduction.

Absorption Depending on Pulse Duration
Once the temperature distribution is obtained, the absorption coefficient and reflectivity are updated based on Eqs. (S4), (S6), and (S10), and light propagation is again calculated. This process is repeated at each time step. The absorbed energy is determined by integrating the absorbed energy density over space and subsequently integrating it over time. The pulse-duration dependence of the energy absorption rate calculated based on this procedure is shown in fig. S4.

Evaluation of the surface damage and filament electron density
To prove that no internal modification occurred inside silica glass after the delivery of a ps Bessel pulse with a pulse energy of 250 μJ, the calculation of laser peak fluence for surface damage evaluation and filament electron density for internal modification evaluation was performed.

Assuming that the intensity distribution of the central lobe of Bessel pulse approximates a Gaussian distribution. The peak fluence of the central lobe can be calculated by $F=2E/(\pi w^2)$, where w is the $1/e^2$ definition of the Gaussian beam radius, E is the total pulse energy of 250 μJ. Based on our experimental results using beam profiler, the full width at half maximum (FWHM) of the central lobe of Bessel pulse is approximately 5.2 μm at the focal plane and the pulse energy of the central lobe accounts for approximately 7.9% of the total pulse energy. The peak fluence of the central lobe is approximately 64.4 J/cm^2, which is larger than the surface ablation

threshold of 10 J/cm^2 with a 10 ps pulse (*62*). Thus, the surface damage will occur after the delivery of the ps laser pulse. As shown in fig. S6, the pump-probe results indicate that after the ps Bessel pulse delivery, i.e., the (*N*-1)th and (*N*-2)th pulses (the laser parameters are the same), small surface damage was observed, which is consistent with the above calculation.
Regarding internal modification, the comparison of the filament free-carrier density with respect to the critical plasma density is applied to evaluate how far the material is from optical breakdown. The estimated electron density inside the filament is obtained from the filament transmission images and calculated according to the Drude model. In the pump-probe experiments, the probe pulse was partially absorbed by the plasma filament. The average absorption coefficient α can be expressed as:

$$\alpha = -\frac{\ln(I_1/I_0)}{d} \tag{S12}$$

where I_1 and I_0 represent the probe intensities after and before passing through the filament, respectively, and d is the diameter of the filament with value of 5 μm. The electron density in the center of the filament is calculated using the following equation (*17*):

$$\rho = \alpha\frac{m_e\varepsilon_0\omega[1+(\omega\tau)^2]}{ke^2\tau} \tag{S13}$$

where m_e is the electron mass, ε_0 is the vacuum dielectric constant, ω is the laser frequency, τ is the collision time of electrons with value of 0.2 fs, and k is the wavenumber.
Fig. S7 shows the electron densities for pump-probe delays of 10 ps and 100 ps. The corresponding filament images are shown in Fig. 2C. The propagation direction of the pump pulse is defined as the depth direction, with the interface between the sample and air set at 0 μm. The maximum electron density in the filament is significantly lower than the critical value of 1.05×10^{27} m^{-3} (*52*). This explains why no noticeable material damage was observed in the filament region, which is also consistent with our experimental results, shown in fig. S6.

Based on the above results, it can be found that after the delivery of a ps Bessel pulse with pulse energy of 250 μJ, a small surface damage and a long channel of transient electronic excitation occurred. The generated filament eventually disappeared, and no permanent internal modification was produced.

Through hole diameter saturation

The saturations in the diameter of drilled through-hole for 1-mm-thick and 0.5-mm-thick silica glass samples were examined by increasing the pulse duration of the μs laser. The ps laser parameters were set at a pulse energy of 250 μJ and a duration of 5 ps.
As shown in Fig. 3 and fig. S10, a wider through-hole with a diameter of approximately 9.5 μm was produced with a pulse duration of 100 μs for the 1-mm-thick silica glass. With a further increase in the μs laser pulse duration, a blind hole was generated due to local thermal absorption of the μs laser energy, although the distance between the bottom of the blind hole and bottom surface was only ~20 μm, as shown in fig. S10B. The diameter of the blind hole extended until reaching saturation at approximately 13.5 μm with a μs laser pulse duration greater than 200 μs. For the 0.5-mm-thick sample, through-holes were consistently generated as the μs laser pulse duration increased. As shown in fig. S11, the diameter of the through-hole increased with increasing μs laser pulse duration until reaching saturation at approximately 15.5 μm for pulse durations exceeding 250 μs. Thus, the drilled through-hole diameter exhibited saturation at

approximately 13.5 μm and 15.5 μm for the 1-mm-thick and 0.5-mm-thick silica glass samples, respectively, using the current ps and μs laser pulse energies.

Evaluation of drilling quality in sapphire and SiC materials

As shown in figs. S21 and S22, the Bessel TSL drilling quality in sapphire and SiC materials is not as good as that in glass materials. In sapphire and SiC, defects can easily modify the optical properties (*63*). During the Bessel TSL machining, defects, such as color-center sites (*64*, *65*) and stacking faults (*66*), are formed inside the materials. This leads to strong absorption and high-temperature plasma formation on the sample surface. These competing undesired phenomena prevent the propagation and absorption of pulse energy inside the samples, resulting in lower machining speed and quality in Bessel TSL machining of these materials compared to glass materials.

Nevertheless, the drilling quality is still superior to that potentially achieved by percussion drilling with conventional ultrashort pulse laser processing. As previously reported (*67*, *68*), a significant amount of damage, such as stress-induced cracks, is generated around the processing area during femtosecond laser processing of SiC. Such damage does not occur during Bessel TSL machining of sapphire and SiC materials, as shown in figs. S21 and S22.

Improvement in the uniformity of the fabricated thousands of through-holes

As shown in Fig. 4B, figs. S6 and S7, some molten materials flow and are ejected during the drilling process, and part of the molten materials are solidified at the entrance area, reducing the uniformity of the fabricated thousands of through-holes, particularly the variance in entrance diameters. This issue is primarily attributed to the uneven thermal fluid behaviors during Bessel TSL machining. The uniformity of the fabricated thousands of through-holes can be improved by further spatially controlling the size of the heating area and material removal region. To achieve this, we used two axicon lenses (AX252, Thorlabs) with a larger base angle of 2°, instead of AX lenses (AX251, Thorlabs) with a base angle of 1°, to generate more confined Bessel core regions for both ps and μs pulses. This can result in more stable thermal fluid dynamics and, consequently, more uniform material removal. Using this setup, we fabricated 50 × 50 through-holes with a pulse energy of 250 μJ for the ps laser and 15 mJ for the μs laser, as well as a pulse duration of 5 ps for the ps laser and 60 μs for the μs laser. As shown in fig. S24, the results indicate that the uniformity, including the variance in entrance diameters, can be controlled to some extent during the large-area glass through-holes fabrication.

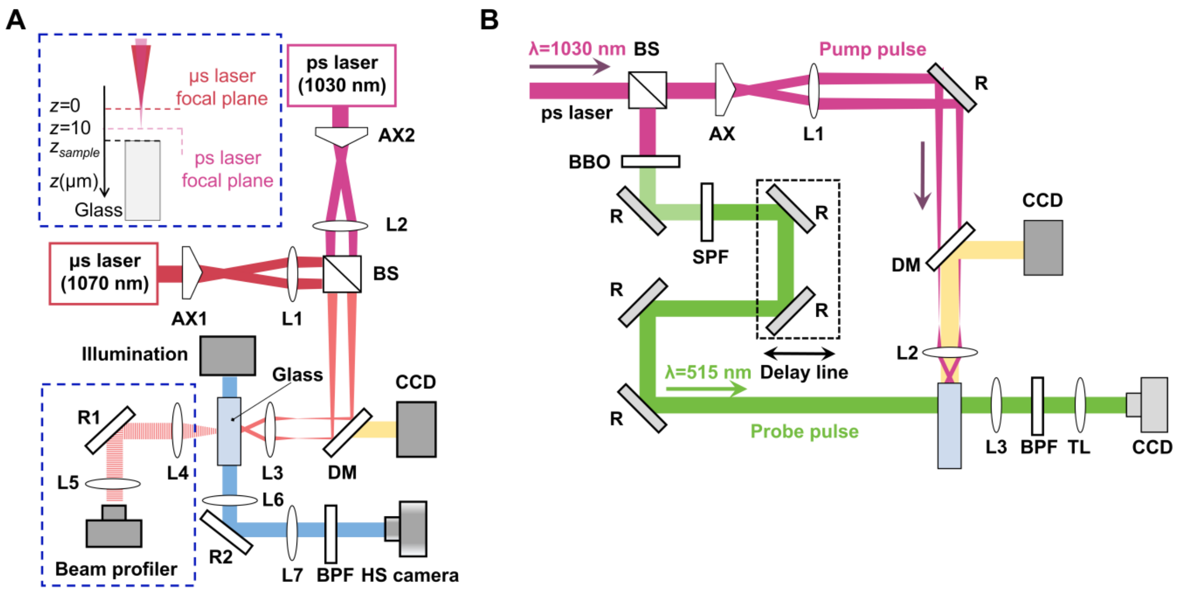

Fig. S1. Schematic of the experimental setup. (**A**) Bessel TSL. AX (AX1 and AX2): Axicon lens; L: lens (L1-L7); BS: beam splitter; DM: dichroic mirror; BPF: band-pass filter; R: reflector; CCD: charge-coupled device. The lower left blue dashed rectangle denotes the setup for pulse measurement using a beam profiler, whereas the upper left blue dashed rectangle denotes the focal planes of the ps and μs lasers. The position of the focal plane of the μs laser is defined as zero position along the z axis. (**B**) Pump-probe. TL: Tube lens; BO: beta barium borate; SPF: short-pass filter; DM: dichroic mirror; BPF: band-pass filter.

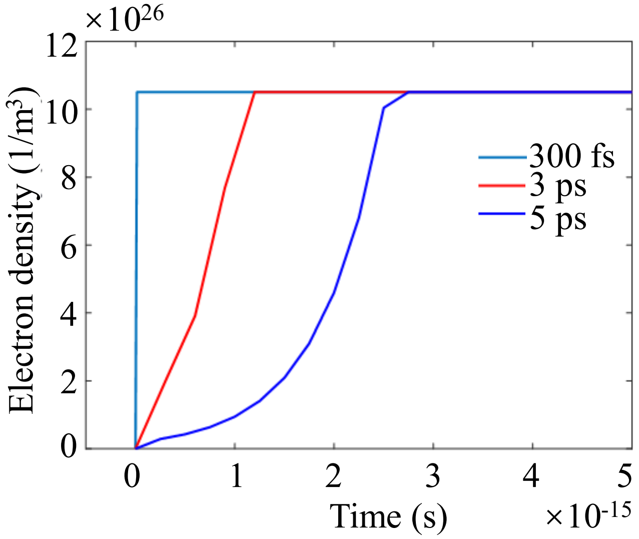

Fig. S2. Variation of the excited electron density on sample surface under different pulse durations of the first pulse. 300 fs, 3 ps and 5 ps indicate the pulse duration of the first pulse. The ps laser pulse energy is 250 μJ.

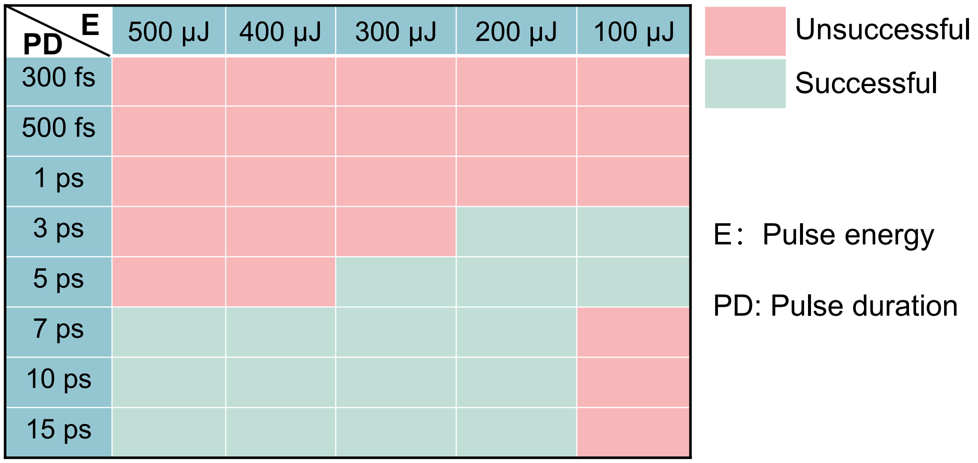

Fig. S3. Effect of the pulse duration and pulse energy of the first pulse on through hole fabrication. Unsuccessful through-holes indicate cases where blind holes or no holes are generated. The pulse energy of 25mJ and pulse duration of 100 μs of μs laser were used.

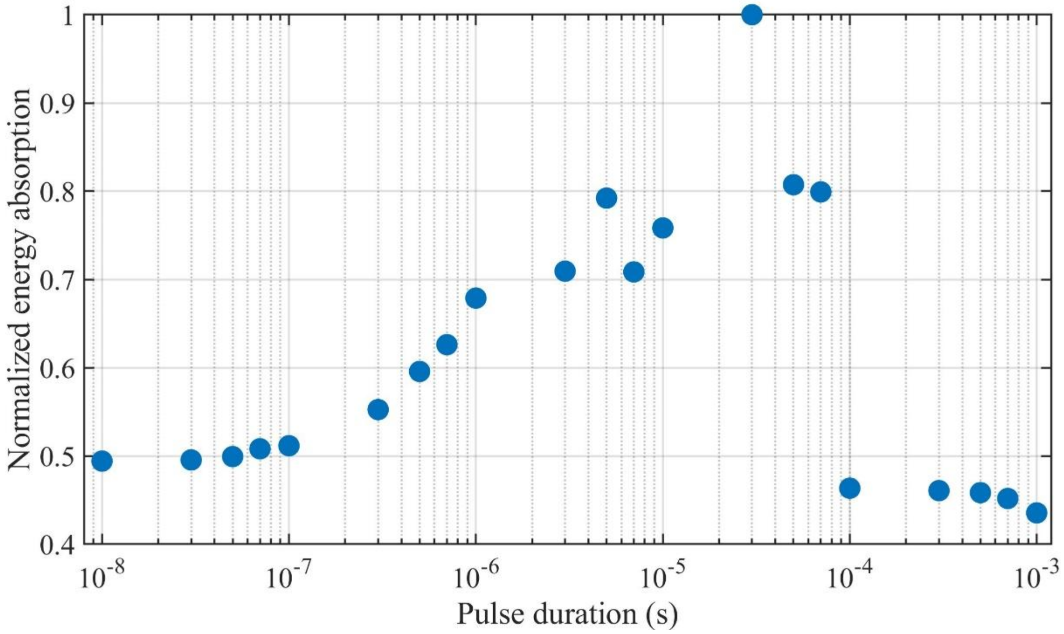

Fig. S4. Effect of the pulse duration of the second pulse on energy absorption. The pulse energy of 250 µJ and pulse duration of 5 ps of the first pulse were used. The pulse energy of the second pulse was 15 mJ.

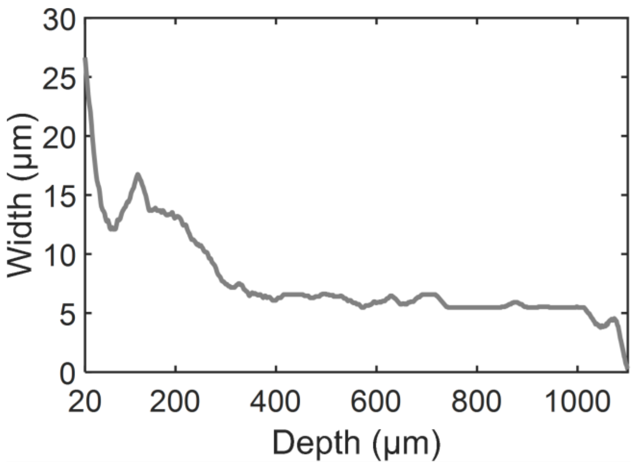

Fig. S5. Filament width changes along depth at delay time of 100 ps. The pulse energy of 250 μJ and pulse duration of 5 ps of the ps pulse were used.

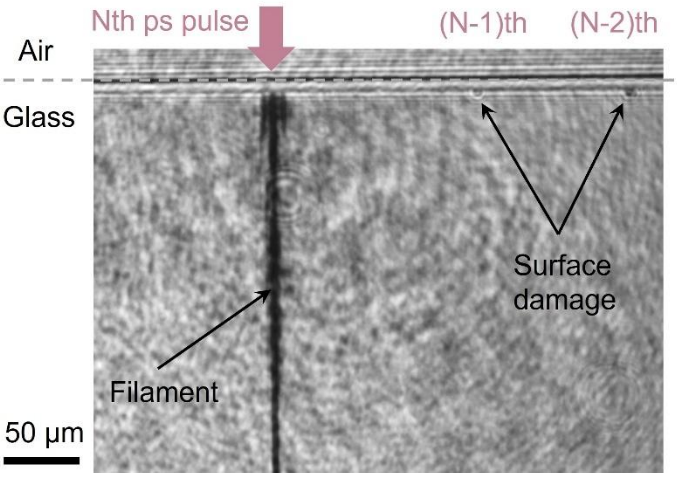

Fig. S6 Pump-probe image under Nth ps Bessel pulse. Pulse energy is 250 μJ and pulse duration is 5 ps. The time delay between the pump and probe pulses is 5 ps.

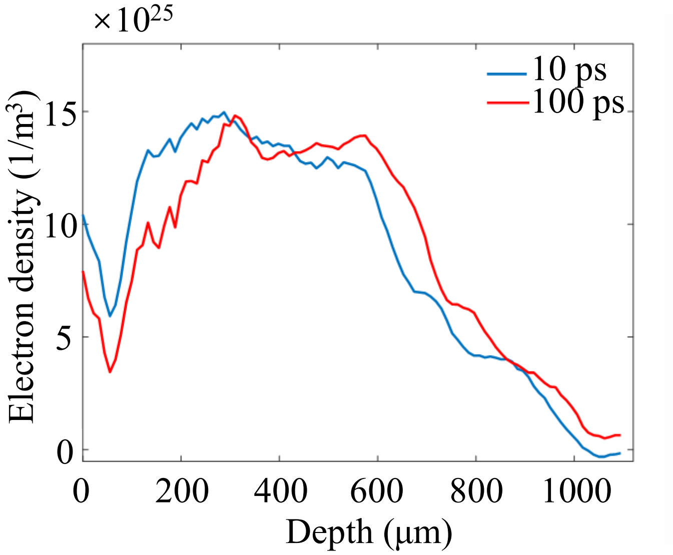

Fig. S7 Distribution of electron density induced by ps pulse. 10 ps and 100 ps indicate the time after the delivery of the ps pulse. The pulse energy of 250 μJ and pulse duration of 5 ps of the ps pulse were used.

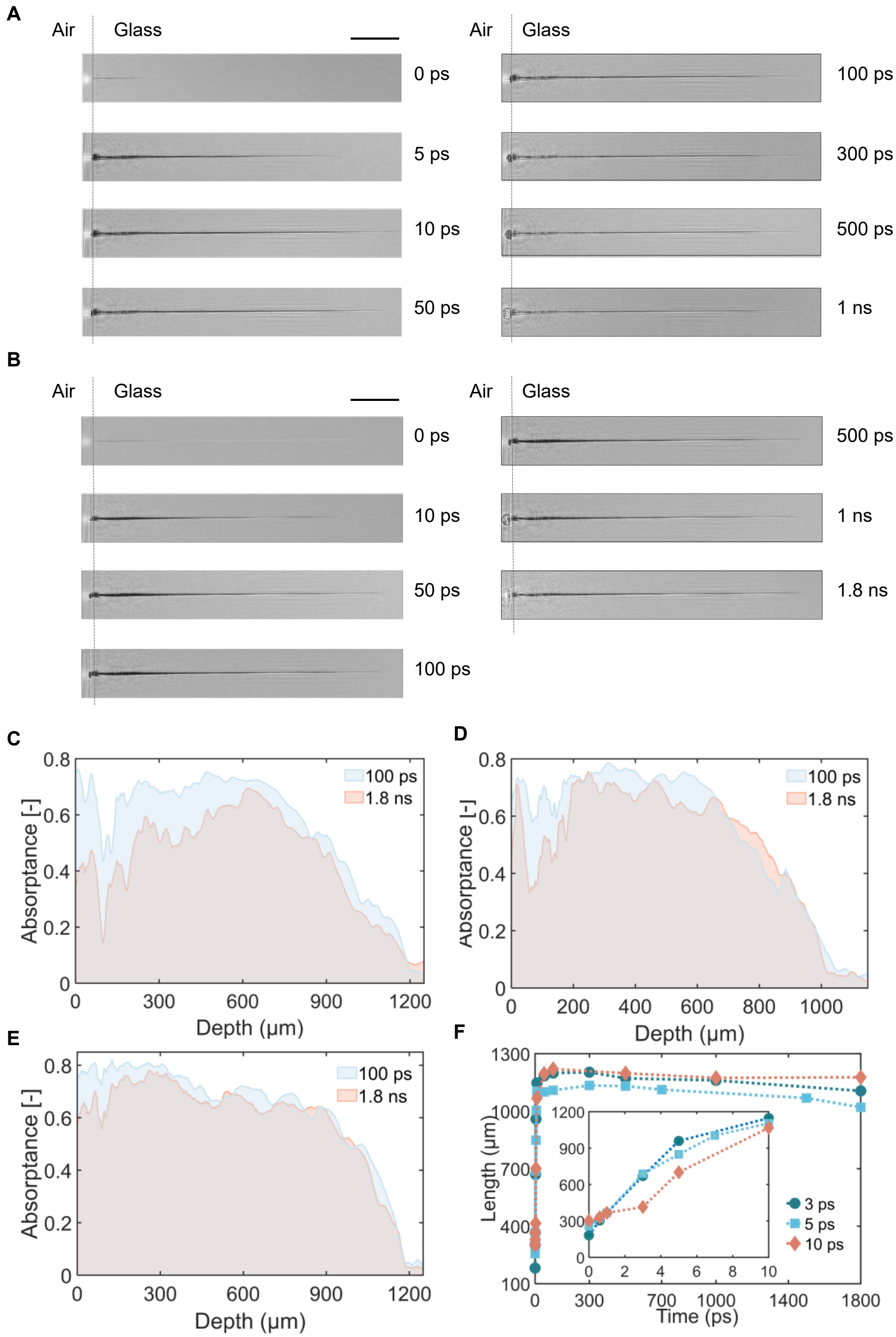

Fig. S8. Filament evolution with different pulse durations of the ps laser. Filament images with pulse durations of (**A**) 3 ps and (**B**) 10 ps. The pulse energy of 250 μJ was used for all experiments. Scale bars denote 200 μm. Variations in absorptance along the depth in 100 ps and

1.8 ns with pulse durations of (**C**) 3 ps, (**D**) 5 ps and (**E**) 10 ps. (**F**) Filament length change over time with different pulse durations. The magnified figure corresponds to the length change within 10 ps.

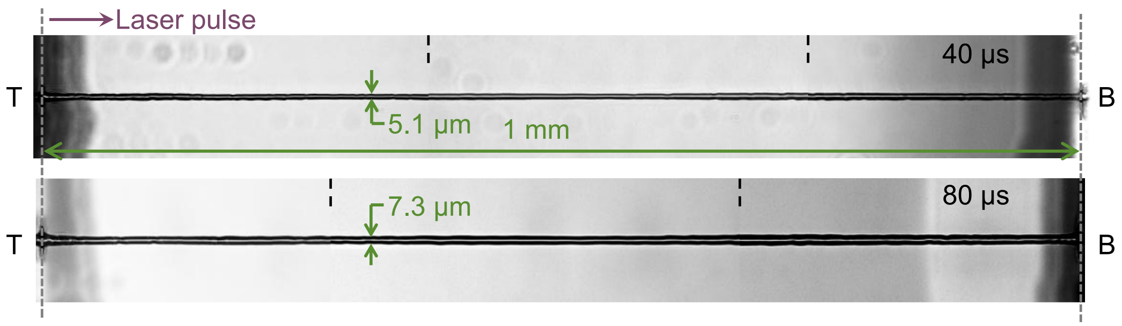

Fig. S9. Full images of drilled through-hole in 1-mm-thick silica glass within 40 μs and 80 μs. The gray dotted line indicates the interface between air and glass. The purple arrow indicates the laser incidence, and the green values indicate the diameters at a depth of 300 μm. Three images captured at different depths were assembled to provide a comprehensive view of the full image. Black dotted lines indicate the borders of the images. T: top surface; B: bottom surface. Scale bars are provided to indicate the thickness of the silica glass. The ps laser parameters were set as a pulse energy of 250 μJ and a pulse duration of 5 ps. The pulse energy of μs laser is 10 mJ and 20 mJ for a pulse duration of 40 μs and 80 μs, respectively.

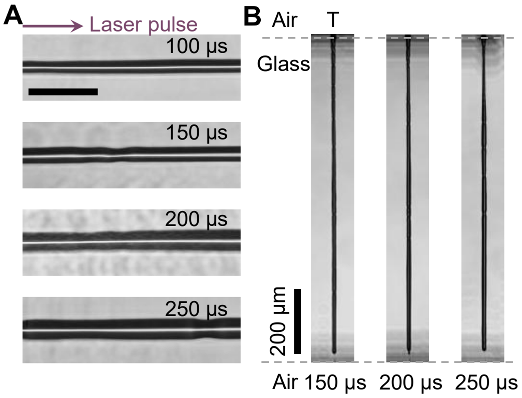

Fig. S10 Variation of the drilled holes in 1-mm-thick silica glass with different pulse durations of µs laser. (**A**) Magnified and (**B**) full images of drilled holes with varying µs laser pulse durations. The scale bar in (A) indicates 50 µm, and T in (B) indicates top surface. Through holes are not generated when the µs laser pulse duration exceeds 100 µs, as shown at the bottom in (B). The ps laser parameters were set as a pulse energy of 250 µJ and a pulse duration of 5 ps.

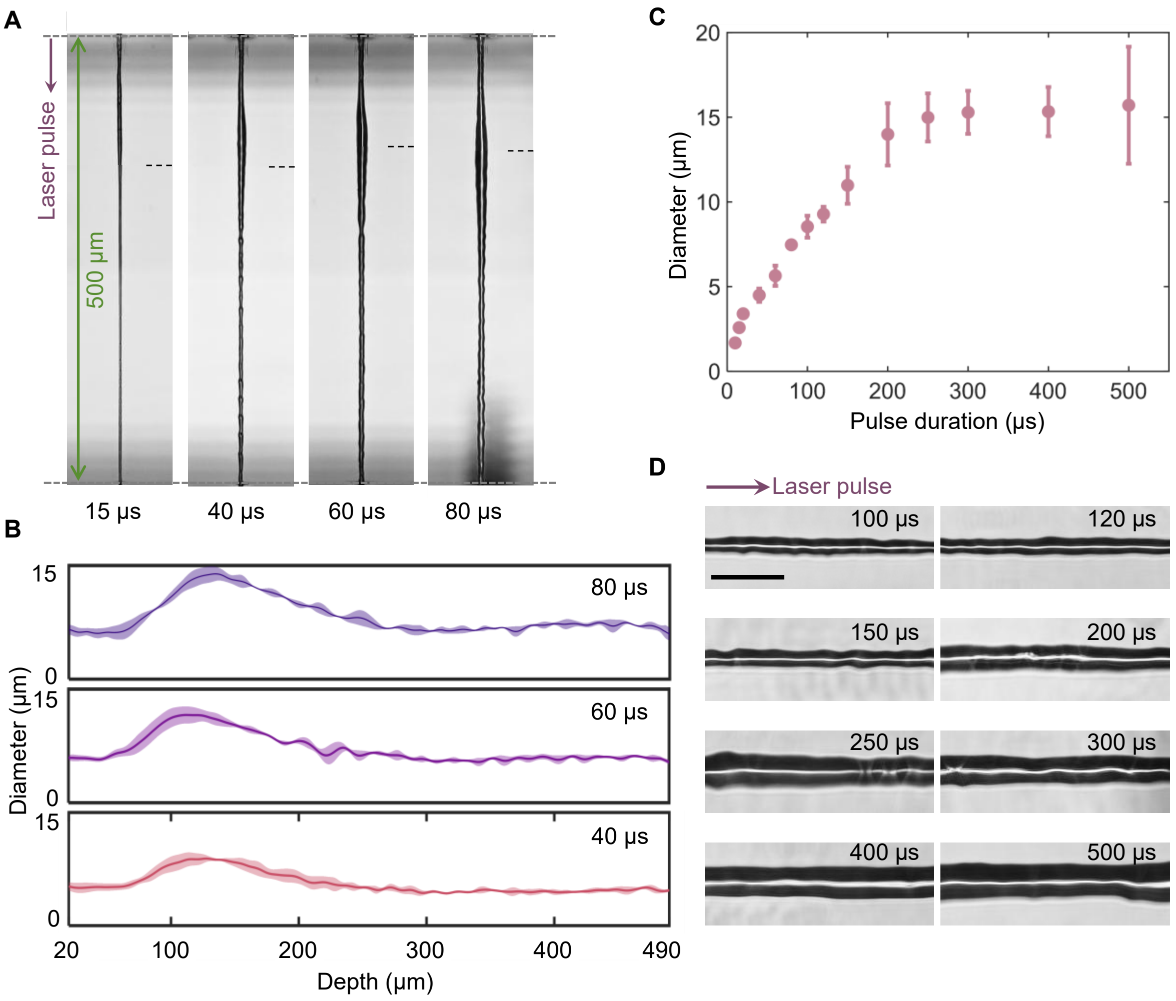

Fig. S11. Variation of the drilled through-holes in 0.5-mm-thick silica glass with different pulse durations of μs laser. (**A**) Full images with different pulse durations of μs laser, i.e. 15 μs, 40 μs, 60 μs and 80 μs. The ps laser parameters were set as a pulse energy of 250 μJ and a pulse duration of 5 ps. Gray dotted line indicates the interface between air and glass. The purple arrow indicates the laser incidence. Two images captured at different depths were assembled to obtain a full image. The black dotted lines indicate the borders of the images. T: top surface. Scale bars are provided to indicate the thickness of silica glass. (**B**) Diameter variation along hole depth with different pulse durations of μs laser. Solid lines represent average values, and the error bars represent the standard deviation with three holes. Diameters near front and back surfaces were excluded owing to low measurement accuracy. (**C**) Diameter variation at a depth of 400 μm for different μs laser pulse durations ranging from 15 μs to 500 μs. (**D**) Magnified images of drilled holes with varying μs laser pulse durations. The scale bar in (D) indicates 50 μm.

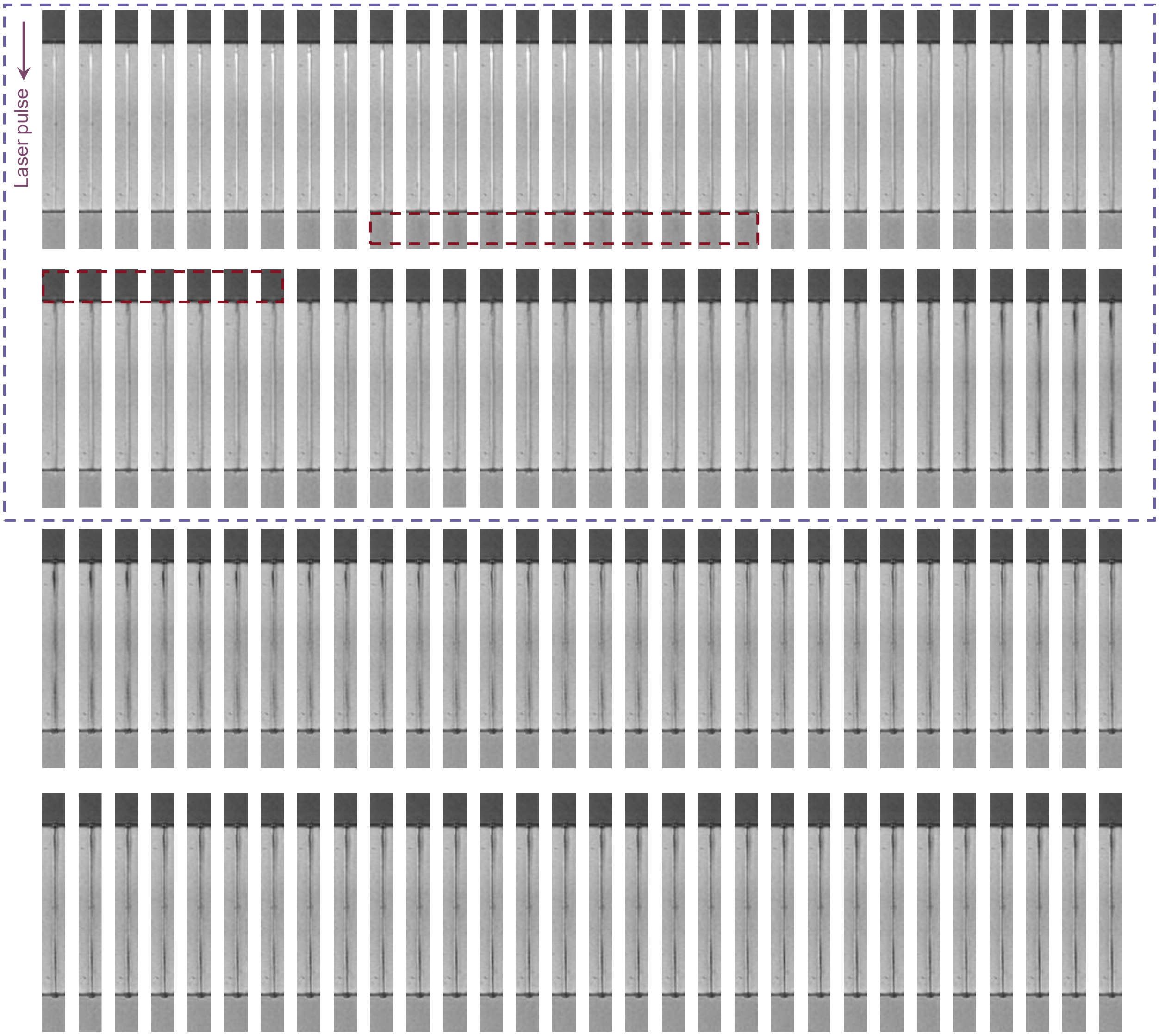

Fig. S12. Time-resolved sequence of drilling a 1-mm-depth through-hole (dark channel) through Bessel TSL. The evolution is monitored over a time domain ranging from 0 μs to 120 μs, where the time interval between images is 1 μs. Two Bessel pulses arrive from the top. Blue dotted rectangle indicates actual drilling time of 60 μs. Red dotted rectangles refer to ejected materials. The pulse energy of 250 μJ for ps laser and 15 mJ for μs laser, as well as the pulse duration of 5 ps for ps laser and 60 μs for μs laser were used.

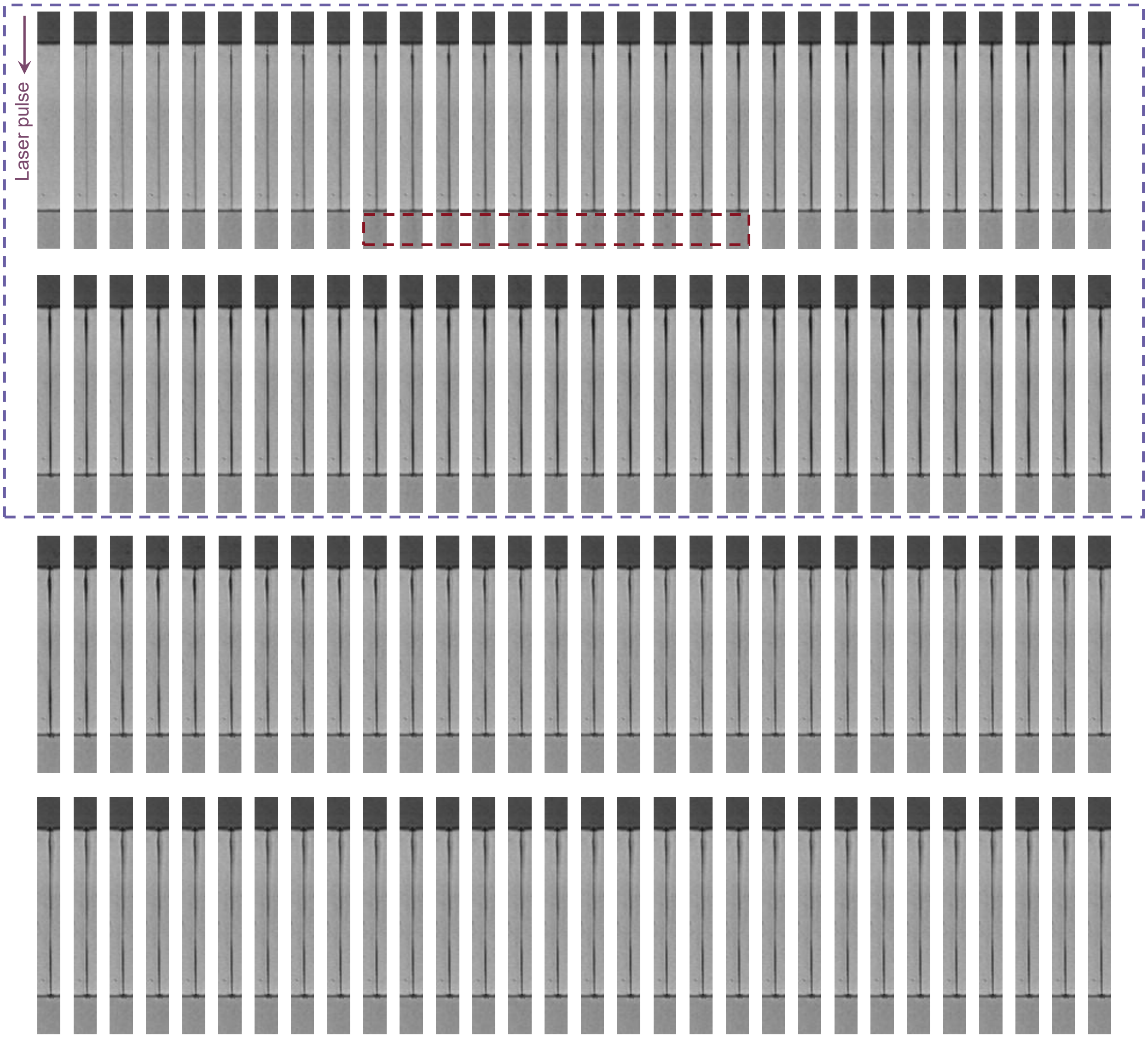

Fig. S13. Time-resolved sequence of drilling a 1-mm-depth through-hole with luminescence through Bessel TSL. The evolution is monitored over a time domain ranging from 0 µs to 120 µs, where the time interval between images is 1 µs. Two Bessel pulses arrive from the top. Blue dotted rectangle represents actual drilling time of 60 µs. Red dotted rectangles refer to ejected materials. The pulse energy of 250 µJ for ps laser and 15 mJ for µs laser, as well as the pulse duration of 5 ps for ps laser and 60 µs for µs laser were used.

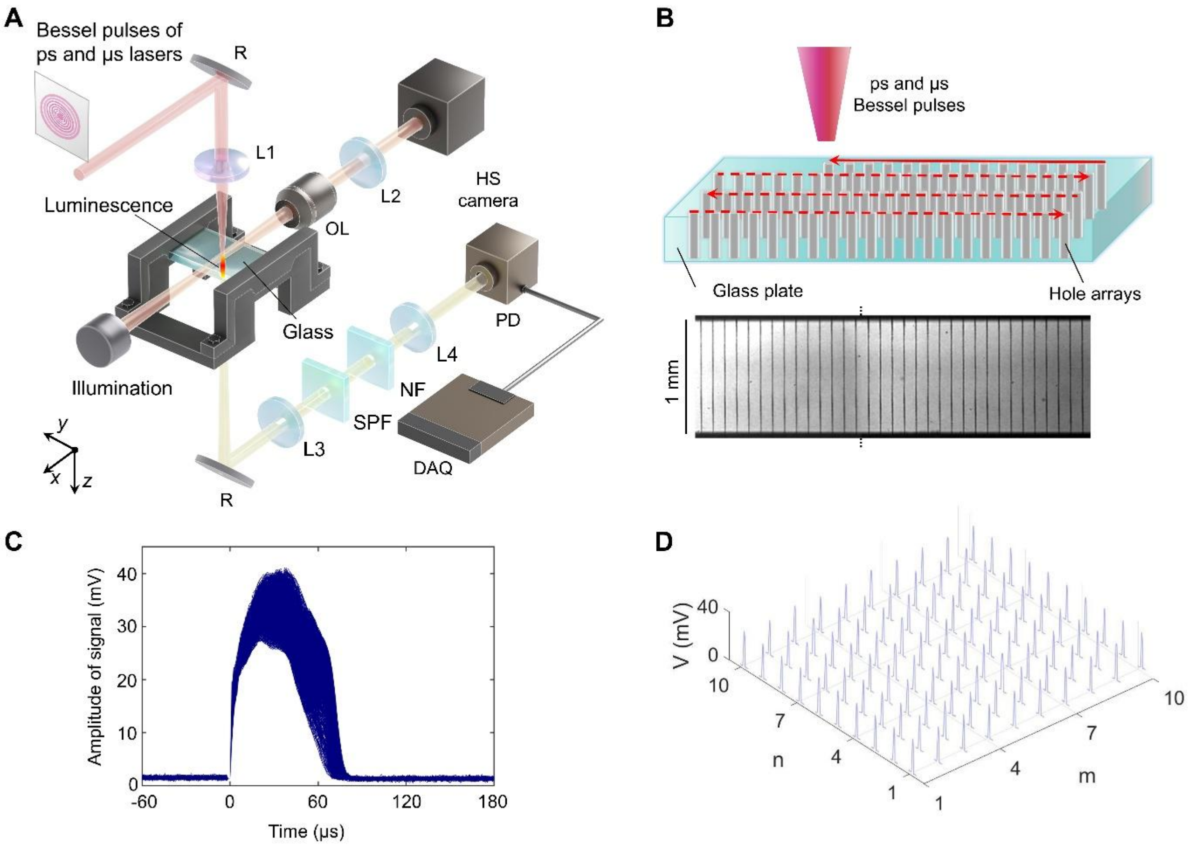

Fig. S14. Fabrication of thousands of through-holes. (A) Schematic of the TGV drilling setup. R: reflector; OL: objective lens; L: lens (L1-L4); NF: notch filter; SPF: short-pass filter; PD: photo detector; DAQ: Data acquisition device; HS camera: high-speed camera. (**B**) Drilling strategy involving a fixed laser spot and continuous moving glass plate. The below image shows the cross-section of multiple holes captured by the high-speed camera. Two images captured at different positions were assembled. The black dotted lines indicate the borders of the images. (**C**) Detected optical signal profiles from luminescence during processing. (**D**) Coordinate map of signals. Each hole has a unique coordinate (*m*, *n*) and corresponding signal profile, used for ultrafast evaluation of hole quality.

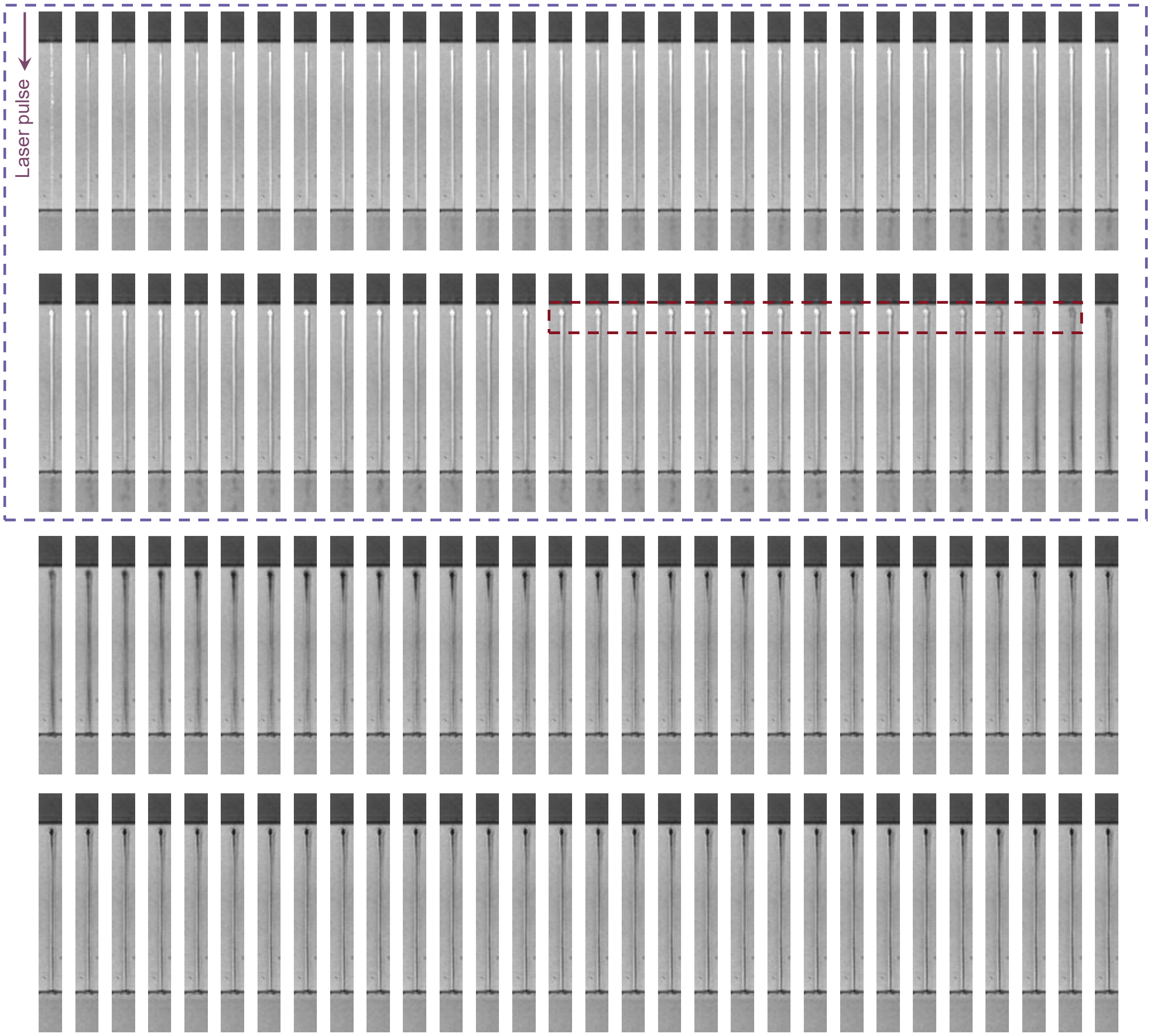

Fig. S15. Time-resolved sequence of drilling a blind hole with luminescence through Bessel TSL. The evolution is monitored over a time domain ranging from 0 µs to 120 µs, where the time interval between images is 1 µs. Two Bessel pulses arrive from the top. Blue dotted rectangle refers to actual drilling time of 60 µs. Red dotted rectangles refer to local absorption and non-penetrated region. The pulse energy of 250 µJ for ps laser and 15 mJ for µs laser, as well as the pulse duration of 5 ps for ps laser and 60 µs for µs laser were used.

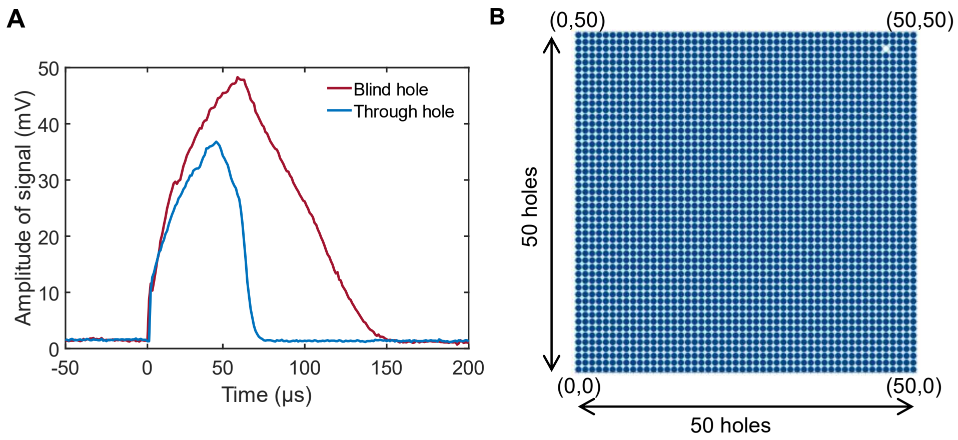

Fig. S16. Evaluation of hole quality. (**A**) Variation curve of optical signal during drilling of through and blind holes with a pulse duration of 60 µs. The pulse energy of 250 µJ for ps laser and 15 mJ for µs laser, as well as a pulse duration of 5 ps for ps laser were used. (**B**) One-to-one mapping for 2500-hole array. The blue circles represent through-holes, whereas the white circle represents blind hole. Each hole has a unique coordinate (*m*, *n*), used for ultrafast evaluation.

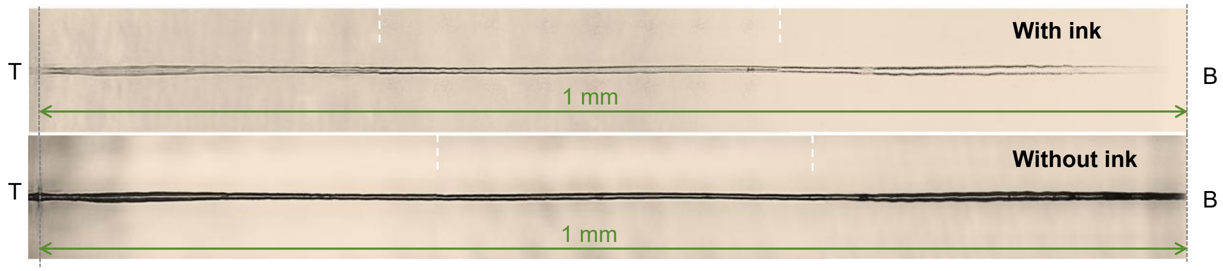

Fig. S17. Microscope image of a 1-mm-depth through-hole with and without filled yellow ink. Three images of each through-hole were assembled to display the full image. The white dotted lines indicate the border. Scale bars indicate the thickness of silica glass. T: top surface; B: bottom surface.

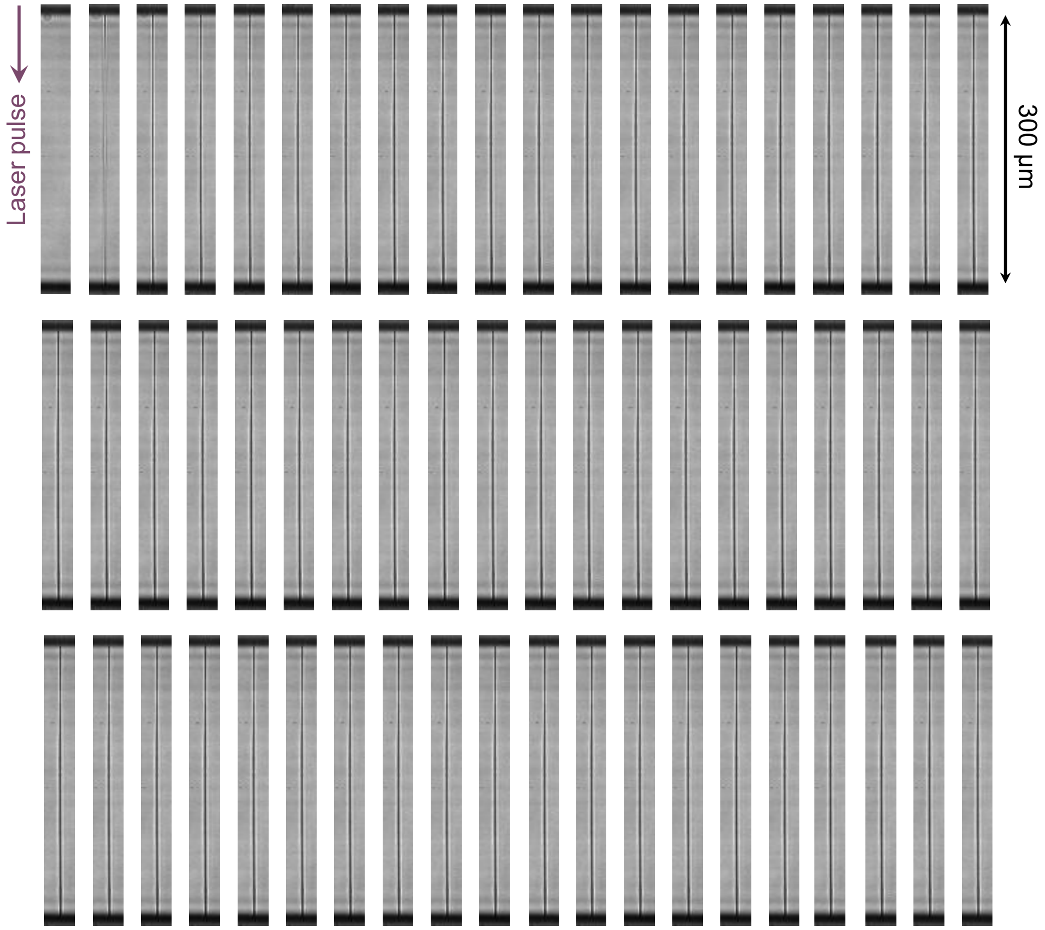

Fig. S18. Time-resolved sequence of drilling a 0.3-mm-thick BK7 glass through Bessel TSL. The evolution is monitored over a time domain ranging from 0 µs to 60 µs, where the time interval between images is 1 µs. Two Bessel pulses arrive from the top. Scale bar indicates the sample thickness of 300 µm. The pulse energy of 250 µJ for ps laser and 15 mJ for µs laser, as well as the pulse duration of 5 ps for ps laser and 60 µs for µs laser were used.

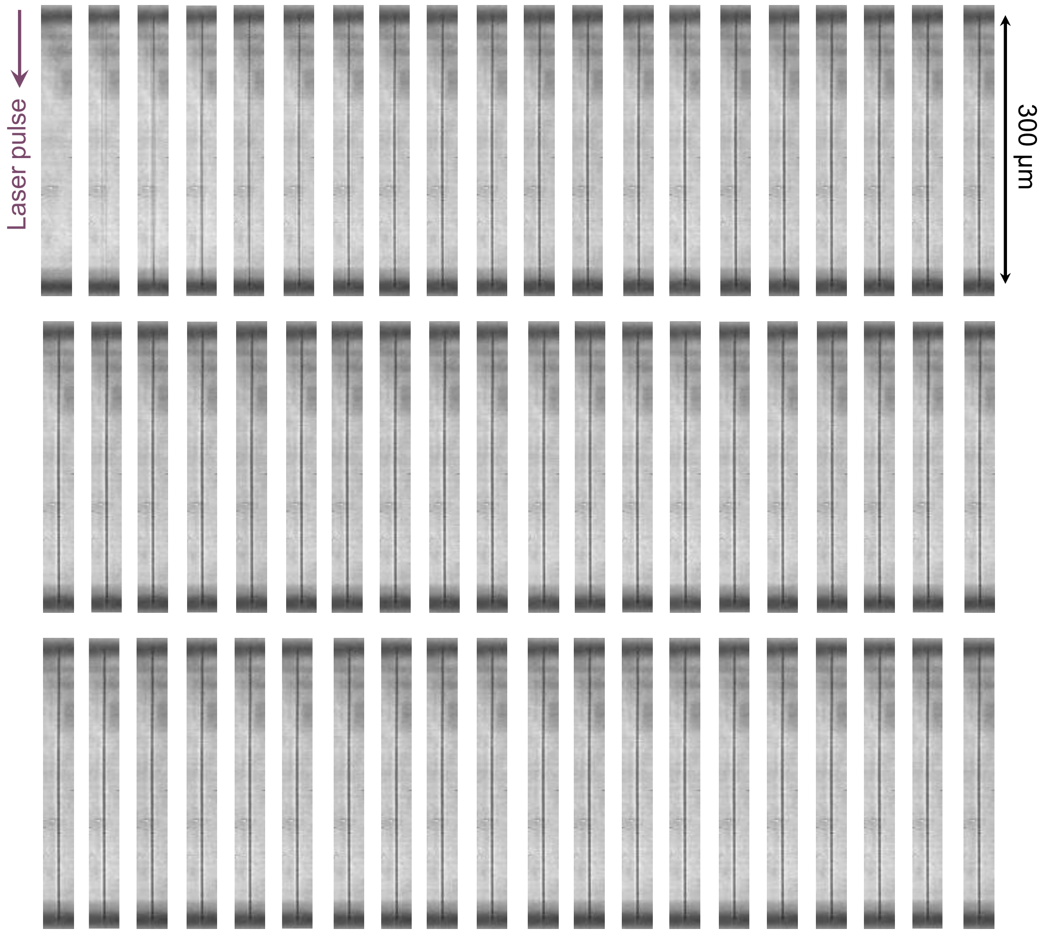

Fig. S19. Time-resolved sequence of drilling a 0.3-mm-thick soda lime glass through Bessel TSL. The evolution is monitored over a time domain ranging from 0 μs to 60 μs, where the time interval between images is 1 μs. Two Bessel pulses arrive from the top. Scale bar indicates the sample thickness of 300 μm. The pulse energy of 250 μJ for ps laser and 15 mJ for μs laser, as well as the pulse duration of 5 ps for ps laser and 60 μs for μs laser were used.

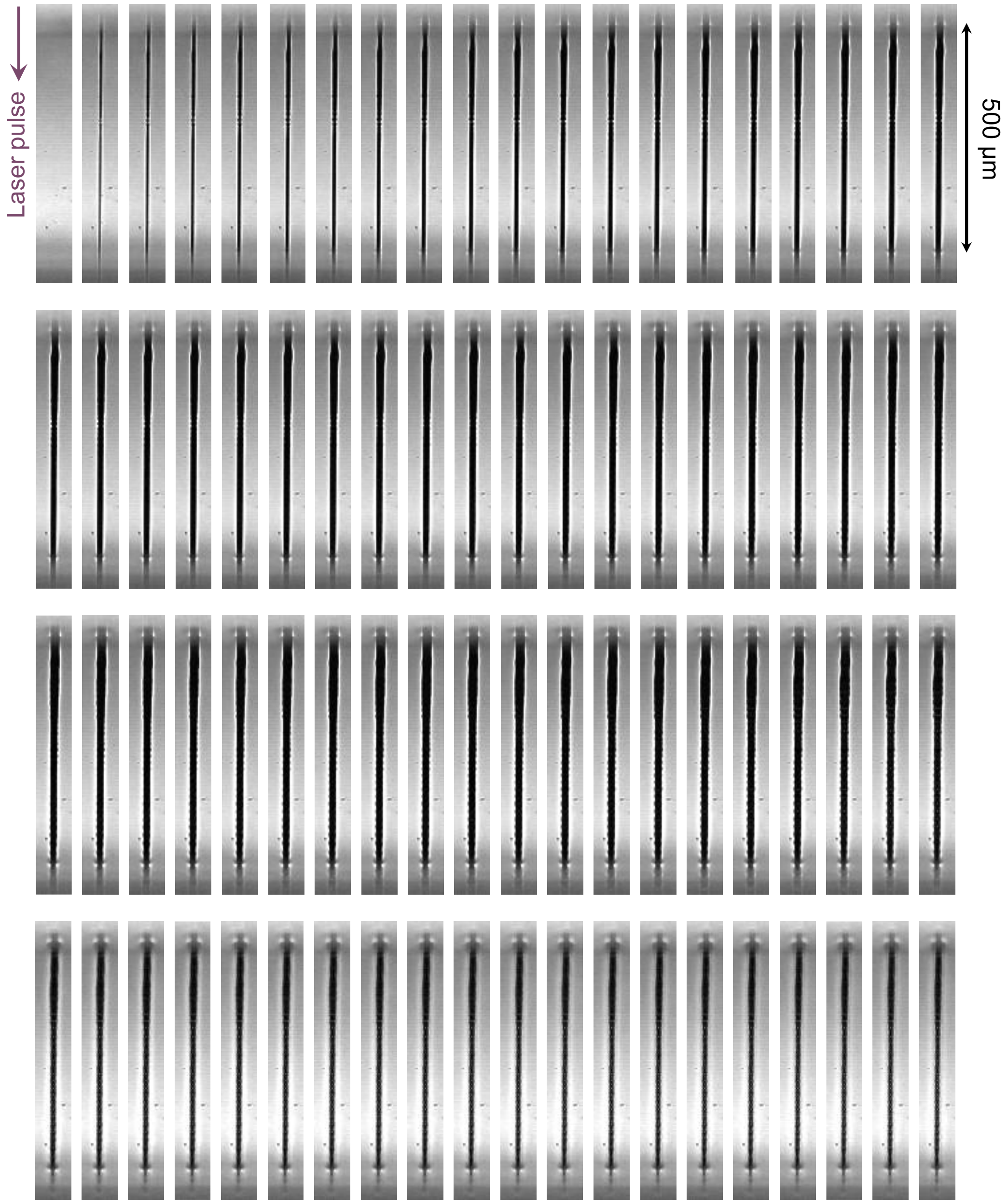

Fig. S20. Time-resolved sequence of drilling a 0.5-mm-thick alkali-free glass through Bessel TSL. The evolution is monitored over a time domain ranging from 0 µs to 80 µs, where the time interval between images is 1 µs. Two Bessel pulses arrive from the top. Scale bar indicates the sample thickness of 500 µm. The pulse energy of 250 µJ for ps laser and 15 mJ for µs laser, as well as the pulse duration of 5 ps for ps laser and 60 µs for µs laser were used.

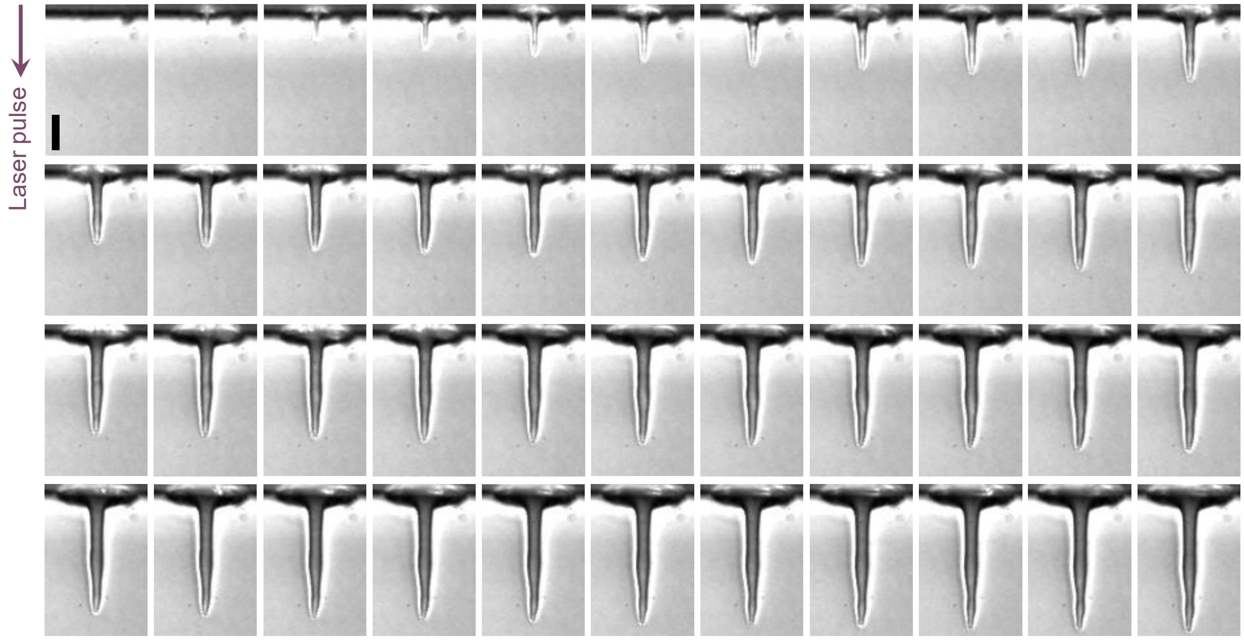

Fig. S21. Time-resolved sequence of drilling a sapphire sample through Bessel TSL. The evolution is monitored over a time domain ranging from 0 μs to 44 μs, where the time interval between images is 1 μs. Two Bessel pulses arrive from the top. Scale bar indicates 100 μm. The pulse energy of 250 μJ for ps laser and 11.2 mJ for μs laser, as well as the pulse duration of 5 ps for ps laser and 45 μs for μs laser were used.

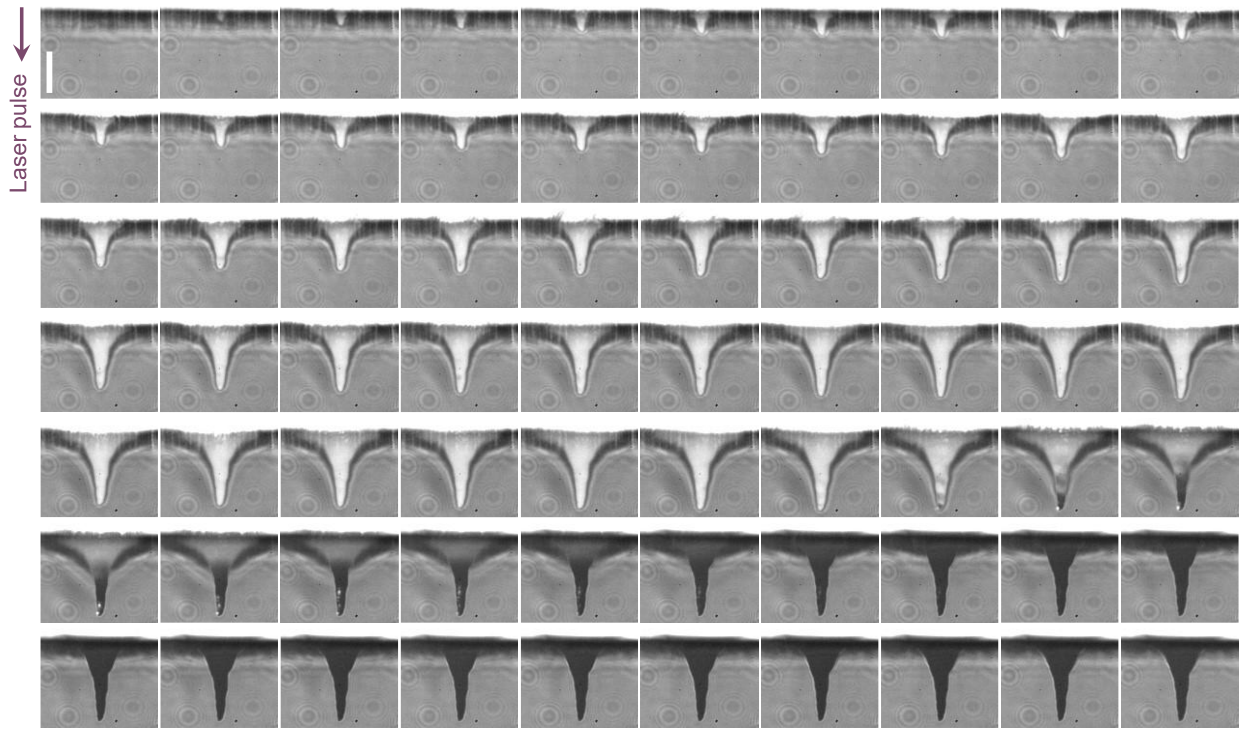

Fig. S22. Time-resolved sequence of drilling a silicon carbide (SiC) sample through Bessel TSL. The evolution is monitored over a time domain ranging from 0 μs to 70 μs, where the time interval between images is 1 μs. Two Bessel pulses arrive from the top. Scale bar indicates 100 μm. The pulse energy of 250 μJ for ps laser and 15 mJ for μs laser, as well as the pulse duration of 5 ps for ps laser and 60 μs for μs laser were used.

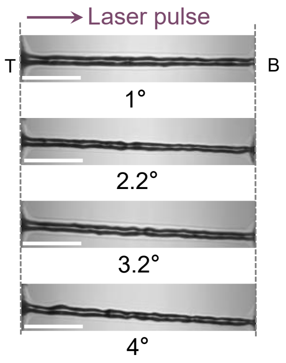

Fig. S23. Microscope images of tilted holes in 0.2-mm-thick silica glass drilled within 60 µs. The scale bar indicates 50 µm. T: top surface; B: bottom surface. The pulse energy of 250 µJ for ps laser and 15 mJ for µs laser, as well as the pulse duration of 5 ps for ps laser and 60 µs for µs laser were used.

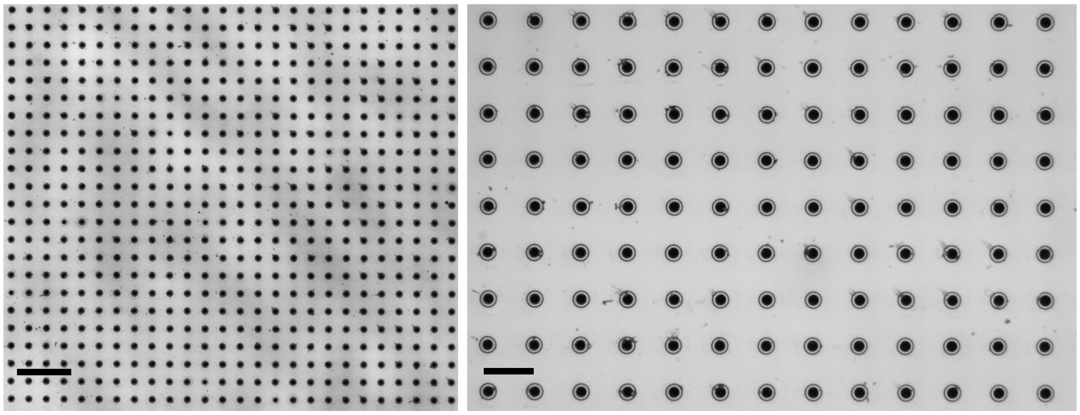

Fig. S24. Microscope images of fabricated through holes using two pairs of axicon lenses with a base angle of 2 °. The scale bar indicates 300 μm in the left image and 100 μm in the right image. The pulse energy of 250 μJ for ps laser and 15 mJ for μs laser, as well as the pulse duration of 5 ps for ps laser and 60 μs for μs laser were used.

Table S1. Physical parameters in the calculation of the energy absorption ratio of the second laser pulse.

Parameters	Value
Refractive index, n_0	1.46
Band gap, U_{gap}	9 eV
Mass density, ρ_{mass}	2.2 g/cm^3
Relaxation time, τ	0.2×10^{-15} s
Specific heat, C_{v}	749 J/(kg·K)
Thermal conductivity, κ	1.38 W/(m·K)

Movie S1. High-speed video of Bessel TSL drilling of silica glass. Frame rate: 1 MHz.

Movie S2. High-speed video of Bessel TSL drilling of BK7 glass. Frame rate: 1 MHz.

Movie S3. High-speed video of Bessel TSL drilling of soda lime glass. Frame rate: 1 MHz.

Movie S4. High-speed video of Bessel TSL drilling of alkali-free glass. Frame rate: 1 MHz.

Movie S5. Actual fabrication video for drilling thousands of holes. 250 holes were drilled per line.

REFERENCES AND NOTES

1. S. Koizumi, K. Watanabe, M. Hasegawa, H. Kanda, Ultraviolet emission from a diamond pn junction. *Science* **292**, 1899–1901 (2001).

2. M. S. Akselrod, F. J. Bruni, Modern trends in crystal growth and new applications of sapphire. *J. Cryst. Growth* **360**, 134–145 (2012).

3. J. Götze, Chemistry textures and physical properties of quartz-geological interpretation and technical application. *Mineral. Mag.* **73**, 645–671 (2009).

4. D. Strickland, G. Mourou, Compression of amplified chirped optical pulses. *Opt. Commun.* **55**, 447–449 (1985).

5. U. Keller, D. A. B. Miller, G. D. Boyd, T. H. Chiu, J. F. Ferguson, M. T. Asom, Solid-state low-loss intracavity saturable absorber for Nd:YLF lasers: An antiresonant semiconductor Fabry–Perot saturable absorber. *Opt. Lett.* **17**, 505–507 (1992).

6. S. Küper, M. Stuke, Femtosecond UV excimer laser ablation. *Appl. Phys. B* **44**, 199–204 (1987).

7. B. Ali, I. V. Litvinyuk, M. Rybachuk, Femtosecond laser micromachining of diamond: Current research status, applications and challenges. *Carbon* **179**, 209–226 (2021).

8. K. Sugioka, Y. Cheng, Femtosecond laser three-dimensional micro- and nanofabrication. *Appl. Phys. Rev.* **1**, 041303 (2014).

9. K. M. Davis, K. Miura, N. Sugimoto, K. Hirao, Writing waveguides in glass with a femtosecond laser. *Opt. Lett.* **21**, 1729–1731 (1996).

10. E. N. Glezer, M. Milosavljevic, L. Huang, R. J. Finlay, T. H. Her, J. P. Callan, E. Mazur, Three-dimensional optical storage inside transparent materials. *Opt. Lett.* **21**, 2023–2025 (1996).

11. F. F. Luo, B. Qian, G. Lin, J. Xu, Y. Liao, J. Song, H. Y. Sun, B. Zhu, J. R. Qiu, Q. Z. Zhao, Z. Xu, Redistribution of elements in glass induced by a high-repetition-rate femtosecond laser. *Opt. Express* **18**, 6262–6269 (2010).

12. A. Marcinkevičius, S. Juodkazis, M. Watanabe, M. Miwa, S. Matsuo, H. Misawa, J. Nishii, Femtosecond laser-assisted three-dimensional microfabrication in silica. *Opt. Lett.* **26**, 277–279 (2001).

13. C. Florea, K. A. Winick, Fabrication and characterization of photonic devices directly written in glass using femtosecond laser pulses. *J. Light. Technol.* **21**, 246–253 (2003).

14. K. Kawamura, M. Hirano, T. Kurobori, D. Takamizu, T. Kamiya, H. Hosono, Femtosecond-laser-encoded distributed-feedback color center laser in lithium fluoride single crystals. *Appl. Phys. Lett.* **84**, 311–313 (2004).

15. J. Zhang, M. Gecevičius, M. Beresna, P. G. Kazansky, Seemingly unlimited lifetime data storage in nanostructured glass. *Phys. Rev. Lett.* **112**, 033901 (2014).

16. K. Sugioka, Y. Hanada, K. Midorikawa, Three-dimensional femtosecond laser micromachining of photosensitive glass for biomicrochips. *Laser Photon. Rev.* **3**, 386–400 (2010).

17. B. C. Stuart, M. D. Feit, S. Herman, A. M. Rubenchik, B. W. Shore, M. D. Perry, Nanosecond-to-femtosecond laser-induced breakdown in dielectrics. *Phys. Rev. B* **53**, 1749–1761 (1996).

18. L. Jiang, A. D. Wang, B. Li, T. H. Cui, Y. F. Lu, Electrons dynamics control by shaping femtosecond laser pulses in micro/nanofabrication: Modeling, method, measurement and application. *Light Sci. Appl.* **7**, 17134–17134 (2018).

19. R. R. Gattass, E. Mazur, Femtosecond laser micromachining in transparent materials. *Nat. Photon.* **2**, 219–225 (2008).

20. D. Tan, B. Zhang, J. Qiu, Ultrafast laser direct writing in glass: Thermal accumulation engineering and applications. *Laser Photon. Rev.* **15**, 2000455 (2021).

21. W. Xiong, Y. S. Zhou, X. N. He, Y. Gao, M. Mahjouri-Samani, L. Jiang, T. Baldacchini, Y. F. Lu, Simultaneous additive and subtractive three-dimensional nanofabrication using integrated two-photon polymerization and multiphoton ablation. *Light Sci. Appl.* **1**, e6 (2012).

22. A. M. Weiner, Femtosecond pulse shaping using spatial light modulators. *Rev. Sci. Instrum.* **71**, 1929–1960 (2000).

23. C. Hnatovsky, R. S. Taylor, E. Simova, V. R. Bhardwaj, D. M. Rayner, P. B. Corkum, Polarization-selective etching in femtosecond laser-assisted microfluidic channel fabrication in fused silica. *Opt. Lett.* **30**, 1867–1869 (2005).

24. Y. Li, K. Itoh, W. Watanabe, K. Yamada, D. Kuroda, J. Nishii, Y. Y. Jiang, Three-dimensional hole drilling of silica glass from the rear surface with femtosecond laser pulses. *Opt. Lett.* **26**, 1912–1914 (2001).

25. C. A. Ross, D. G. MacLachlan, D. Choudhury, R. R. Thomson, Optimisation of ultrafast laser assisted etching in fused silica. *Opt. Express* **26**, 24343–24356 (2018).

26. J. Dudutis, L. Zubauskas, E. Daknys, E. Markauskas, R. Gvozdaitė, G. Račiukaitis, P. Gečys, Quality and flexural strength of laser-cut glass: Classical top-down ablation versus water-assisted and bottom-up machining. *Opt. Express* **30**, 4564–4582 (2022).

27. M. K. Bhuyan, F. Courvoisier, P. A. Lacourt, M. Jacquot, R. Salut, L. Furfaro, J. M. Dudley, High aspect ratio nanochannel machining using single shot femtosecond Bessel beams. *Appl. Phys. Lett.* **97**, 081102 (2010).

28. M. Duocastella, C. B. Arnold, Bessel and annular beams for materials processing. *Laser Photon. Rev.* **6**, 607–621 (2012).

29. P. Balage, J. Lopez, G. Bonamis, C. Hönninger, I. Manek-Hönninger, Crack-free high-aspect ratio holes in glasses by top–down percussion drilling with infrared femtosecond laser GHz-bursts. *Int. J. Extreme Manuf.* **5**, 015002 (2022).

30. C. Kerse, H. Kalaycıoğlu, P. Elahi, B. Çetin, D. K. Kesim, Ö. Akçaalan, S. Yavaş, M. D. Aşık, B. Öktem, H. Hoogland, R. Holzwarth, F. Ö. Ilday, Ablation-cooled material removal with ultrafast bursts of pulses. *Nature* **537**, 84–88 (2016).

31. K. Sugioka, M. Iida, H. Takai, K. Midorikawa, Efficient microwelding of glass substrates by ultrafast laser irradiation using a double-pulse train. *Opt. Lett.* **36**, 2734–2736 (2011).

32. Y. Ito, R. Yoshizaki, N. Miyamoto, N. Sugita, Ultrafast and precision drilling of glass by selective absorption of fiber-laser pulse into femtosecond-laser-induced filament. *Appl. Phys. Lett.* **113**, 061101 (2018).

33. Y. Kawasuji, J. Fujimoto, M. Kobayashi, A. Suwa, A. Mizutani, M. Arakawa, H. Mizoguchi, Deep ultraviolet excimer laser processing for the micro via hole on semiconductor package. *J. Laser Appl.* **32**, 022076 (2020).

34. L. Rapp, R. Meyer, R. Giust, L. Furfaro, M. Jacquot, P. A. Lacourt, J. M. Dudley, F. Courvoisier, High aspect ratio micro-explosions in the bulk of sapphire generated by femtosecond Bessel beams. *Sci. Rep.* **6**, 34286 (2016).

35. M. K. Bhuyan, P. K. Velpula, J. P. Colombier, T. Olivier, N. Faure, R. Stoian, Single-shot high aspect ratio bulk nanostructuring of fused silica using chirp-controlled ultrafast laser Bessel beams. *Appl. Phys. Lett.* **104**, 021107 (2014).

36. R. Meyer, L. Froehly, R. Giust, J. Del Hoyo, L. Furfaro, C. Billet, F. Courvoisier, Extremely high-aspect-ratio ultrafast Bessel beam generation and stealth dicing of multi-millimeter thick glass. *Appl. Phys. Lett.* **114**, 201105 (2019).

37. F. He, J. Yu, Y. Tan, W. Chu, C. Zhou, Y. Cheng, K. Sugioka, Tailoring femtosecond 1.5-µm Bessel beams for manufacturing high-aspect-ratio through-silicon vias. *Sci. Rep.* **7**, 40785 (2017).

38. J. del Hoyo, R. Meyer, L. Furfaro, F. Courvoisier, Nanoscale confinement of energy deposition in glass by double ultrafast Bessel pulses. *Nanophotonics* **10**, 1089–1097 (2021).

39. G. Ren, Y. Ito, H. Sun, N. Sugita, Temporal-spatial characteristics of filament induced by a femtosecond laser pulse in transparent dielectrics. *Opt. Express* **30**, 4954–4964 (2022).

40. P. Polesana, A. Couairon, D. Faccio, A. Parola, M. A. Porras, A. Dubietis, A. Piskarskas, P. Di Trapani, Observation of conical waves in focusing, dispersive and dissipative Kerr media. *Phys. Rev. Lett.* **99**, 223902 (2007).

41. Q. Sun, H.-B. Jiang, Y. Liu, Y.-H. Zhou, H. Yang, Q.-H. Gong, Relaxation of dense electron plasma induced by femtosecond laser in dielectric materials. *Chin. Phys. Lett.* **23**, 189–192 (2006).

42. F. Huang, J. Si, T. Chen, T. Shen, M. Shi, X. Hou, Temporal-spatial dynamics of electronic plasma in femtosecond laser induced damage. *Opt. Express* **29**, 14658–14667 (2021).

43. T. C. Zhu, V. V. Gunaratne, M. Lozovoy, M. Dantus, In-situ femtosecond laser pulse characterization and compression during micromachining. *Opt. Express* **15**, 16061–16066 (2007).

44. Y. Zhang, Y. Ito, R. Yoshizaki, A. Shibata, I. Nagasawa, K. Nagato, N. Sugita, Mechanism and performance evaluation of transient and selective laser processing of glass based on optical monitoring. *Opt. Express* **31**, 38191–38204 (2023).

45. T. Li, H. Xu, M. Panmai, T. Shao, G. Gao, F. Xu, S. Zhu, Ultrafast metaphotonics. *Ultrafast Sci.* **4**, 0074 (2024).

46. D. Puerto, W. Gawelda, J. Siegel, J. Bonse, G. Bachelier, J. Solis, Transient reflectivity and transmission changes during plasma formation and ablation in fused silica induced by femtosecond laser pulses. *Appl. Phys. A* **92**, 803–808 (2008).

47. T. Okuchi, M. Takigawa, J. Shu, H.-K. Mao, R. J. Hemley, T. Yagi, Fast molecular transport in hydrogen hydrates by high-pressure diamond anvil cell NMR. *Phys. Rev. B* **75**, 144104 (2007).

48. A. O. Watanabe, T. H. Lin, M. Ali, Y. Wang, V. Smet, P. M. Raj, M. Swaminathan, Ultrathin antenna-integrated glass-based millimeter-wave package with through-glass vias. *IEEE Trans. Microw. Theory Technol.* **68**, 5082 (2020).

49. Y. Jia, Y. Xu, Y. Guo, A universal scalable thermal resistance model for compact large-signal model of AlGaN/GaN HEMTs. *IEEE Trans. Microw. Theory Tech.* **66**, 4419–4429 (2018).

50. H. Tanaka, D. Iwata, Y. Shibata, T. Hase, D. Onoshima, N. Yogo, H. Shibata, M. Sato, K. Ishikawa, I. Nagasawa, Y. Hasegawa, M. Ishii, Y. Baba, M. Hori, High-performance glass

filters for capturing and culturing circulating tumor cells and cancer-associated fibroblasts. *Sci. Rep.* **13**, 4130 (2023).

51. P. K. Kennedy, A first-order model for computation of laser-induced breakdown thresholds in ocular and aqueous media. I. Theory. *IEEE J. Quantum Electron.* **31**, 2241–2249 (1995).

52. A. Vogel, J. Noack, G. Hüttman, G. Paltauf, Mechanisms of femtosecond laser nanosurgery of cells and tissues. *Appl. Phys. B* **81**, 1015–1047 (2005).

53. M. K. Bhuyan, F. Courvoisier, P. A. Lacourt, M. Jacquot, L. Furfaro, M. J. Withford, J. M. Dudley, High aspect ratio taper-free microchannel fabrication using femtosecond Bessel beams. *Opt. Express* **18**, 566–574 (2010).

54. T. Koike, N. Sugita, Y. Ito, Experimental investigation and modeling of spatio-temporal dynamics of filamentary regions induced by an ultrashort laser pulse. *Opt. Express* **32**, 35268–35286 (2024).

55. M. D. Perry, B. C. Stuart, P. S. Banks, M. D. Feit, V. Yanovsky, A. M. Rubenchik, Ultrashort-pulse laser machining of dielectric materials. *J. Appl. Phys.* **85**, 6803–6810 (1999).

56. M. Lamperti, V. Jukna, O. Jedrkiewicz, P. Di Trapani, R. Stoian, T. E. Itina, C. Xie, F. Courvoisier, A. Couairon, Invited article: Filamentary deposition of laser energy in glasses with Bessel beams. *APL Photonics* **3**, 120805 (2018).

57. M. K. Bhuyan, P. K. Velpula, M. Somayaji, J.-P. Colombier, R. Stoian, 3D nano-fabrication using controlled Bessel-glass interaction in ultra-fast modes. *J. Laser Micro Nanoeng.* **12**, 274–280 (2017).

58. I. H. Chowdhury, X. Xu, A. M. Weiner, Ultrafast double-pulse ablation of fused silica. *Appl. Phys. Lett.* **86**, 151110 (2005).

59. G. Ren, Y. Ito, R. Yoshizaki, H. Sun, J. Hattori, N. Sugita, Ultrafast dynamics and internal processing mechanism of silica glass under double-pulse femtosecond laser irradiation. *Opt. Express* **32**, 32408–32420 (2024).

60. A. Vogel, B. Rockwell, “Roles of tunneling, multiphoton ionization, and cascade ionization for femtosecond optical breakdown in aqueous media” (Tech. Rep., Lubeck Medical Univ. Medical Laser Center, 2009).

61. R. Yoshizaki, Y. Ito, S. Yoshitake, C. Wei, A. Shibata, I. Nagasawa, K. Nagato, N. Sugita, Mechanism of material removal through transient and selective laser absorption into excited electrons in fused silica. *J. Appl. Phys.* **130**, 053102 (2021).

62. D. Nieto, J. Arines, G. M. O’Connor, M. T. Flores-Arias, Single-pulse laser ablation threshold of borosilicate, fused silica, sapphire, and soda-lime glass for pulse widths of 500 fs, 10 ps, 20 ns. *Appl. Optics* **54**, 8596–8601 (2015).

63. H. Jo, Y. Ito, J. Hattori, K. Nagato, N. Sugita, High-speed observation of damage generation during ultrashort pulse laser drilling of sapphire. *Opt. Commun.* **495**, 127122 (2021).

64. A. C. Tam, J. L. Brand, D. C. Cheng, W. Zapka, Picosecond laser sputtering of sapphire at 266 nm. *Appl. Phys. Lett.* **55**, 2045–2047 (1989).

65. S. Castelletto, A. F. M. Almutairi, K. Kumagai, T. Katkus, Y. Hayasaki, B. C. Johnson, S. Juodkazis, Photoluminescence in hexagonal silicon carbide by direct femtosecond laser writing. *Opt. Lett.* **43**, 6077–6080 (2018).

66. U. Rehman, K. A. Janulewicz, Structural transformations in femtosecond laser-processed *n*-type 4H-SiC. *Appl. Surf. Sci.* **385**, 1–8 (2016).

67. J. Hattori, Y. Ito, K. Nagato, N. Sugita, Investigation of damage generation process by stress waves during femtosecond laser drilling of SiC. *Precis. Eng.* **72**, 789–797 (2021).

68. H. Sun, Y. Ito, G. Ren, J. Hattori, K. Nagato, N. Sugita, Observation of damage generation induced by electron excitation and stress wave propagation during ultrashort pulse laser drilling of sapphire. *Appl. Phys. A* **128**, 547 (2022).
